# Tau profiling of brain extracellular vesicles reveals PHF6 peptide as core for pathological tau seeding in Alzheimer's disease

**DOI:** 10.1186/s12929-026-01250-1

**Published:** 2026-06-15

**Authors:** Marie Oosterlynck, Laura Fichter, Elodie Leroux, Sabiha Eddarkaoui, Thomas Bouillet, Camille Lefebvre, Marine Nguyen, Claude-Alain Maurage, Bertrand Accart, Elian Dupré, Isabelle Landrieu, Clément Danis, Sylvain Lehmann, Jérôme Vialaret, Luc Buée, Christophe Hirtz, Morvane Colin

**Affiliations:** 1 University of Lille, Inserm, CHU Lille, U1172 - LilNCog - Lille Neuroscience & Cognition, 59000 Lille, France; 2 CNRS, EMR9002 - BSI - Integrative Structural Biology, 59000 Lille, France; 3 University of Lille, Inserm, CHU Lille, Pasteur Institute of Lille, U1167 - RID-AGE - Risk Factors and Molecular Determinants of Aging-Related Diseases, 59000 Lille, France; 4 University of Lille, CHU-Lille, CRB/CIC1403, Centre de Ressources Biologiques du Centre d’Investigation Clinique, 59000 Lille, France; 5 IRMB-PPC, University of Montpellier, INM Inserm, CHU Montpellier, 34000 Montpellier, France

**Keywords:** Tauopathies, Alzheimer’s disease, Extracellular Vesicles, Seeding, Tau proteoforms, PHF6 peptide, Ubiquitination

## Abstract

**Background:**

Tauopathies are neurodegenerative diseases all characterized by tau lesions in the brain. Nevertheless, a clinical and pathophysiological heterogeneity is present among them. This includes the dominant tau isoform found within aggregates (3R and/or 4R tau) along with different brain regions being affected. For some tauopathies, especially in Alzheimer’s disease, a specific spatio-temporal staging of tau lesions is present. This staging has been the basis for the prion-like propagation hypothesis, which describes a cell-to-cell transfer of pathological tau species resulting in new aggregates formation in recipient neurons. Human extracellular vesicles isolated from the brain-derived fluid (BD-EVs) of Alzheimer’s disease patients contain seeds that contribute to this tau pathology spreading. However, the nature of these tau species responsible for this nucleation activity remains unknown. Additionally, heterogeneity in seeding activity of BD-EVs of Alzheimer’s disease, progressive supranuclear palsy and Pick’s disease patients is known.

**Methods:**

Here, EVs were isolated from human frozen tissue (Alzheimer’s disease, Progressive Supranuclear Palsy, Pick disease and non-demented controls). We used a tau immunoprecipitation followed by high-resolution mass spectrometry to define their proteomic profile and test their seeding capacity in vitro.

**Results:**

We show that the tau profile present within BD-EVs is different among tauopathies. Interestingly, multiple tau peptides located in the microtubule binding region were specifically enriched in Alzheimer’s disease extracellular vesicles. Of these, mainly the PHF6 (VQIVYK) containing proteins mediate tau seeding activity.

**Conclusions:**

PHF6 is a driver for the higher EVs-mediated tau propagation in AD patients, revealing an interesting therapeutic target to prevent tau pathology spreading.

**Supplementary Information:**

The online version contains supplementary material available at 10.1186/s12929-026-01250-1.

## Background

The microtubule-associated tau protein is an intrinsically disordered protein that plays many different roles in cells [1–4]. In the brain, it is composed of four domains: the N-terminal domain, the proline rich region (PRR), the microtubule-binding region (MTBR) and the C-terminal domain. It can undergo alternative splicing of exon 2 and/or exon 3 that generates three N-isoforms (0N, 1N and 2N). Likewise, an alternative splicing of exon 10 located in repeat domain 2 (RD2) of the MTBR can occur, resulting in two R-isoforms (3R and 4R). In total, this results in six tau isoforms in the adult human brain[5]. Aside from different isoforms, tau is also known to undergo post-translational modifications (PTMs) such as phosphorylation, methylation, ubiquitination, glycosylation, acetylation and many more [6–9]. In pathological conditions, the tau protein can be found hyper- and abnormally phosphorylated. This has led to the hypothesis that these PTMs may be at the origin of a structural change in the tau protein that leads to its aggregation [10, 11].

Tauopathies is an umbrella term for a group of heterogeneous neurodegenerative brain disorders all characterized by the inclusion of tau aggregates within the cells. The tauopathy with the highest world prevalence is Alzheimer’s disease (AD), which is characterized by intracellular tau aggregates along with extracellular amyloid**-**ß plaques and therefore considered as a secondary tauopathy. Numerous primary tauopathies have also been described such as Progressive Supranuclear Palsy (PSP) and Pick’s disease (PiD) [12–14].

Although all tauopathies comprise aggregated tau, the tau lesions do not result in similar clinical deficits [15]. This clinical heterogeneity may be the consequence of the heterogeneity observed in numerous pathological aspects. First, the dominant isoforms of tau found within the aggregates are different leading to a post-mortem histopathological stratification of tauopathies. AD including both 3R and 4R tau is referred to as 3R/4R-tauopathy, while PSP is referred to as a 4R-tauopathy and PiD as a 3R-tauopathy [6, 14]. Secondly, the aggregates adopt different three-dimensional structures [16–18]. Thirdly, these lesions are described in different cell types of the brain [15]. Additionally, some tauopathies have a unique spatio-temporal staging based on the anatomical occurrence of tau lesions. For AD, these are described as the well-characterized Braak stages, which detail a tau pathology sequential evolution from the entorhinal cortex to the hippocampus, to finally invade the associative and primary neocortex [19–21]. A less robust hierarchical staging in PSP evolves from the pallido-luyso-nigral complex to the frontal and parietal lobes [22–25] and in PiD, it evolves through the limbic and frontotemporal neocortical regions [26]. The robust Braak stages in AD insinuate a cell-to-cell spreading of pathological tau, driving the prion-like tau propagation [27, 28]. For this, cytosolic translated tau is believed to be transported in the extracellular space through unconventional protein secretion (UPS) mechanisms, including extracellular vesicles (EVs) [29].

EVs are bilipid spheres known for their role in cellular communication through transport of proteins, lipids, RNA and more [30]. In the brain, EVs are directly released in the interstitial fluid surrounding neural cells and hence entitled brain-derived EVs (BD-EVs). Human BD-EVs of AD patients contain seed-competent tau [31–33]. Further, we demonstrated a different tau seeding capacity of AD, PSP and PiD patients-derived EVs. This reflects a heterogeneous contribution of BD-EVs to tau seeding among tauopathies, with AD BD-EVs showing the highest seeding capacity [32]. Here, we aim to identify and compare the tau proteoforms found within EVs of tauopathies to assess if this tau content lays at the base of the heterogeneity observed for EVs seeding among tauopathies. A tau immunoprecipitation followed by mass spectrometry analysis (tau IP-MS) performed on BD-EVs from non-demented controls (CTRL) or patients affected with different tauopathies, revealed detection of 22 tryptic tau peptides with two MTBR tryptic peptides enriched only in AD BD-EVs. Among them, the well-known pro-aggregative hexapeptide PHF6 (VQIVYK) was found [34–37]. Importantly, we demonstrated that PHF6-containing tau proteins are strongly involved in the seeding capacity of AD BD-EVs, where PHF6-ubiquitination is also part of it. From this, we conclude that the PHF6 is a driver for the higher EVs-mediated tau propagation in AD patients, revealing an interesting therapeutic target to prevent tau pathology spreading.

## Methods

### Human samples

The cohort in this study has been used in prior study published in Leroux and collaborators [32] and comprises of CTRL, AD, PSP and PiD fresh-frozen brain extracts obtained from the Lille Neurobank (fulfilling French legal requirements concerning biological resources and declared to the competent authority under the number DC2008-642) with donor consent, data protection and Ethics Committee approval. Samples were managed by the CRB/CIC1403 Biobank, BB-0033-00030. Neuropathological assessment of tau pathology was previously quantified using AT8 immunostaining in the prefrontal cortex, showing that tau burden was highest in AD cases, followed by PiD and then PSP [32]. The prefrontal cortex (Brodmann area 9) was selected in this study for BD-EV isolation, as we previously showed a tau seeding capacity of prefrontal BD-EVs across all three tauopathies studied here [32]. A summary of the demographic data is listed in Table 1.

**Table 1:** Demographic, biological and clinical characteristics of the human brain sample donors

Patient ID	Sex	Age of death (y)	PMD (h)	Diagnosis	Braak stage	Thal stage	Cause of death
A	M	78	19	CTRL	0	0	Invasive aspergillosis
B	M	23	24	CTRL	0	0	Myocarditis
C	M	59	13	CTRL	0	0	Septic shock
D	M	41	11	CTRL	0	0	Suffocation
E	M	70	30	AD	VI	4	
F	F	63	15	AD	VI	4	
G	F	60	24	AD	VI	5	
H	F	82	84	AD	VI	5	
I	F	87	24	AD	VI	5	
J	F	71	4	AD	VI	4	
K	M	64	20	AD	VI	4	
L	M	66	27	AD	VI	5	
M	F	66	16	AD	VI	4	
N	M	69	6	AD	VI	4	
O	M	69	17	PSP	N/A	0	
P	M	82	4	PSP	N/A	0	
Q	M	65	18	PSP	N/A	0	
R	F	79	4	PSP	N/A	0	
S	F	77	9	PSP	N/A	3	
T	M	65	18	PSP	N/A	0	
U	M	84	24	PSP	N/A	0	
V	M	73	9	PSP	N/A	1	
W	M	58	20	PSP	N/A	1	
X	M	57	22	PiD	N/A	0	
Y	M	71	21	PiD	N/A	3	
Z	M	68	15	PiD	N/A	0	
AA	M	68	8	PiD	N/A	0	

CTRL (n = 4), AD patients (AD, n = 10), Progressive Supranuclear Palsy patients (PSP, n = 9) and Pick’s Disease patients (PiD, n = 4) used for brain-derived fluid (BDF) preparation and EVs isolation. *PMD* post-mortem-delay, *N/A* not applicable

### Brain-derived fluid isolation

To obtain the BDF, either papain or collagenase were used for the dissociation of the frozen human prefrontal brain extracts. Papain enzyme was used for brain dissociation to obtain the BD-EVs samples for tau-enriched mass spectrometry analysis. This was performed as previously described [38] and adapted in our previous study [32]. Briefly, frozen brain tissue (60 mg for proteomic analysis for each patient) was incubated on ice in Hibernate-A (50 mM NaF, 200 nM Na3VO4, 10 nM protease inhibitor (E64 from Sigma)) and then gently homogenized in a Potter before adding papain enzyme (2 mL of 20 units/mL; LS003119, Worthington). After a 20-min (min) incubation at 37 °C on wheel, 15 mL of cold Hibernate-A and protease inhibitor cocktail (Roche) were added and mixed by inversions to stop the enzymatic activity.

BD-EVs prepared for functional assays, involving immunodepletion (ID) and immunocapture (IC) with analysis of the seeding capacity on the fluorescence resonance energy transfer (FRET)-tau biosensor cells, were prepared using collagenase type 3 as this will only target the ECM and hence preserves EVs integrity [39]. The protocol using collagenase type 3 (LS0004182, Pan Biotech) for enzymatic tissue dissociation, was performed as previously described by Vella and collaborators [40]. Briefly, brain tissue (60 mg from a pool of three AD patients) was sliced on ice to generate smaller sections (~ 2 mm) before adding 75 units/mL of collagenase type 3 in Hibernate-E (800 μL per 100 mg of tissue, 10315538, Gibco). After an incubation of 20 min at 37 °C with agitation, PhosSTOP (4906837001, Roche) and Complete Protease Inhibitor including EDTA (4693124001, Roche) were added to a final 1X concentration on ice.

For both protocols, successive centrifugations of 300 × g for 5 min, 2000 × g for 10 min and 10,000 × g for 30 min at 4 °C were applied to remove cells, membranes and debris, respectively. The final supernatant is entitled BDF and was consistently prepared fresh prior to each EVs isolation.

### Brain-derived EVs (BD-EVs)

To isolate EVs from the BDF and to separate them from proteins contaminants, size-exclusion chromatography (SEC) was used as described previously by Leroux and collaborators [32]. Briefly, commercial SEC columns (ICO-70, IZON) packed with Sepharose resin CL-2B (CL2B300, Sigma-Aldrich) were equilibrated with degassed phosphate-buffered saline (PBS, 12559069, Gibco), 500 µL of BDF were applied on the SEC column followed by elution in degassed PBS. Once the void volume (3 mL) was eluted, the first 2 mL (F1-4) were recovered as EVs fraction in protein low binding tubes (0030108132, Eppendorf protein LoBind). We previously characterized this fraction enriched in BD-EVs in accordance with MISEV guidelines [32, 41].

One SEC was performed per individual patient for tau IP-Ms and from a pool of three AD patients (patient ID: F, K and M in Table 1) for ID and IC assays. The 2 mL EVs fraction were concentrated using ultrafiltration device 3 kDa Amicon (Amicon® Ultra-2 3 kDa, Millipore) at 4000 × g (Multifuge X3R, Thermo Scientific) to a final volume of 50 µL/SEC/patient or 200 µL/SEC for IP-MS or ID/IC assays, respectively. Concentrated EVs were quantified by NTA.

### Nanoparticle tracking analysis (NTA)

The concentration of particles was measured by NTA (NanoSight NS300, Malvern Panalytical) immediately after isolation. For this, BD-EVs samples were diluted in PBS and continuously infused into the NTA device by an automatic syringe pump at a flow rate of 20 μL/min. The focus was adjusted and the temperature was set to 25 °C. Three videos of 60 s were acquired at camera level 15 and processed at detection level 4 using the NTA software [v 3.2.16]. Samples were freshly used for IC and ID experiments and only EVs designated for proteomic analysis were stored at − 20 °C before analysis.

### Total tau Simoa protein quantification

The BD-EVs from one SEC purification were concentrated using ultrafiltration device 3 kDa Amicon (Amicon® Ultra-15 3 kDa, UFC900324, Millipore) to 400 µL for EVs. Prior to loading, on the Simoa plate, all samples were centrifuged at 10,000 × g for 5 min at RT to remove debris and a 1/1000 dilution was done with the provided dilution buffer. The Simoa Neurology 4-Plex B assay (N4PB, 103,345, Quanterix) was used to measure the concentration total tau on the Simoa® HD-X Analyzer, following the manufacturer’s instructions. All standards, controls and samples were run in simplicate. Concentrations of the target proteins were calculated using a four-parameter logistic (4PL) standard curve generated by the Quanterix software and were adjusted based on the sample dilution. The lower limit of quantification for tau is 0.5 pg/mL.

### Anti-tau antibody production and biotinylation

Antibodies used for the tau enrichment for IP-MS were home-made. Mice received injections every three-weeks of tau peptides with sequences 30–44 (for anti-Tau Exon 1 (TauE1C1)), 162–175 (for anti-TauPRR (TauP1)) or 427–441 (for anti-TauCter (Tau7F5)) coupled to KLH to amplify mouse immunisation. After four injections, lymphoid cells isolated from the spleen were immortalized by fusion with immortal myeloma cells SP2/0 in the presence of PEG/DMSO to generate hybridomas. Culture medium containing 15% of fetal bovine serum, 2 mM L-glutamine and 50 U/mL penicillin/streptomycin was used. For hybridoma selection, hypoxanthine aminopterin thymidine was added for 1 month following the protocol of Köhler and collaborators [42]. Clonal selection was done based on ELISA affinity to 1N4R recombinant tau protein. After two subcloning, clones were amplified. Monoclonal cells were cultured in CELLine flask (WCL1000—3, WHEATON) in Hybridoma-SFM (12045084, Gibco) complemented with 2 mM L-glutamine (25030081, Gibco) and 50 U/mL penicillin/streptomycin. Culture medium containing monoclonal antibodies was collected every 3 to 4 days and filtered using a Filtropur PES 0.45 µm membrane (83.1826, Sarstedt). The antibodies isotype was determined using the SBA ClonotypingTM System/HRP (5300-05, SouthernBiotech, 1/500 dilution in PBS) following manufacturers’ instructions and monoclonal antibodies were purified using affinity chromatography. For this, the automated ÄKTAprime plus (Cytiva) was coupled to a HiTrap HP column coated with protein A or G (17-0404-01 or 17-0402-01, Cytiva) and was used with the chromatogram profile obtained at absorbance 280 nm. First, column equilibration was done in filtered and degassed 20 mM sodium phosphate pH 7. A volume of 30 mL of supernatant from cell culture in CELLine flask with monoclonal antibodies was loaded and elution was done in 0.1 M glycine pH 2.7 where the pH of the antibody samples was directly neutralized in 1 M Tris buffer pH 9. Ten fractions of 500 µL were collected. Antibody containing fractions were selected after electrophoresis using a 1 h (h) Coomassie brilliant blue staining (161-0406, Biorrad, 0.1% Blue G250 in 50% ethanol and 10% acetic acid) on denaturant NuPAGE gels (Thermofisher). Two subsequent dialysis of selected antibody fractions were done for storage in PBS. Antibody concentrations were measured using the NanoDrop™ One (ThermoFisher) at 280 nm. Antibody epitopes were verified by nuclear magnetic resonance (NMR) by comparing spectra of ^15^N 2N4R tau in presence and absence of the antibody. These results along with the validation of detection of human tau for the three home-made tau antibodies is shown in supplementary Fig. 1.

The produced TauE1C1, TauP1 and Tau7F5 antibodies were biotinylated using the EZ link Sulfo-NHS-SS-biotinylation kit (21445, Thermo Fisher) following the protocol recommendations of the manufacturer. Briefly, 2 mg antibody in 1 mL filtered PBS were incubated with 10 mM biotin on a wheel at low speed at 4 °C for 2 h in protein low binding Eppendorf tubes. After this, the biotinylated antibody was separated from unbound biotin using 5 mL Zeba Spin Desalting columns (7 K MWCO, 89981, Thermo Fisher). The eluate containing the antibody-biotin complex was collected and concentrations were measured using the NanoDrop™ One (Thermo Fisher). Biotinylation was validated using ELISA against recombinant 1N3R tau protein (Supplementary Fig. 2A, B). Biotinylated antibodies were stored at 4 °C.

### ^15^N-labeled recombinant tau

Stable isotope–labeled recombinant tau (^15^N-tau) isoforms (0N3R, 1N4R and 2N4R) were used for mass spectrometry (1) to enable alignment of tau peptide *m/z* peaks, (2) to allow absolute quantification of tau peptides and (3) to facilitate identification of tau post-translational modifications. Because samples were digested with trypsin, three recombinant isoforms were sufficient to generate peptides representative of all isoform (0N, 1N, 2N, 3R and 4R) variants and to achieve comprehensive peptide coverage across tau isoforms. These ^15^N-labeled recombinant tau were generated as described previously by Danis and collaborators [43]. Briefly, the pET15b-Tau recombinant T7 plasmids were transformed into competent E. coli BL21 (DE3) bacterial cells of which a small-scale culture was grown in Luria–Bertani medium at 37 °C. For production of recombinant ^15^N-labeled tau0N4R, tau1N3R and tau2N4R, the small-scale culture was cultured in 1 L of a modified M9 medium containing MEM vitamin mix 1 × (Sigma-Aldrich), 4 g of glucose, 1 g of ^15^N-NH_4_Cl (Sigma-Aldrich), 0.5 g of ^15^N-enriched Isogro Growth Powder (Sigma-Aldrich), 0.1 mM CaCl_2_, and 2 mM MgSO_4_. Once the culture reached an optical density of 0.8 at 600 nm, 0.5 mM isopropyl-β-D-thiogalactopyranoside was added to induce expression of ^15^N-recombinant tau. Next, the cells were lysed in 50 mM NaPi pH 6.5 with 2.5 mM EDTA, complete protease inhibitor cocktail (Sigma-Aldrich). The tau proteins were first purified by heating the bacterial extract for 15 min at 75 °C using a water bath. After centrifugation, the resulting supernatant was then passed on a cation exchange chromatography column (CEX, Hitrap SP Sepharose FF, 5 ml, Cytiva) equilibrated in 50 mM NaPi pH 6.5 and eluted with a NaCl gradient. A buffer exchange with 50 mM ammonium bicarbonate (Hiload 16/60 desalting column, Cytiva) of the recombinant tau proteins was done before lyophilization. ^15^N-labeled recombinant tau was suspended to 1 mg/ml in 50 mM ammonium bicarbonate and 1 mM bovine serum albumin. The solutions were diluted at 100 µg/mL and then aliquoted into LoBind tubes and stored at − 80 °C until use to avoid thawing cycles.

### BD-EVs tau enrichment and proteomic sample preparation

BD-EVs samples (2 × 10^10^ particles) from individual patients were incubated with MB2 solution (40 mM Tris–HCl, 137 mM NaCl, 2 mM EDTA, 10 mM Guanidine and 2% IGEPAL), 20 ng of ^15^N-labeled recombinant tau (8 ng 2N4R, 6 ng 1N3R and 6 ng 0N4R) and a combination of three home-made biotinylated monoclonal anti-tau antibodies (2 µg TauE1C1, 1 µg TauP1 and 2 µg Tau7F5), with homogenization performed after each addition. Samples were then incubated overnight at 4 °C with shaking. The added value of tau enrichment using a combination of three tau antibodies, targeting distinct regions of the protein, is demonstrated in Supplementary Fig. 2C-D. Tau immunocapture was performed by the AssayMAP Bravo platform (Agilent Technologies) using Steptavidin cartridges and the "Affinity purification" program. The sample was eluted with 25 µL of elution buffer (12 mM NaCl, 100 mM HCl, pH 2) and then neutralized with 20 µL of 100 mM Tris–HCl at pH 8.5. The eluate was digested in a mix of 1 M urea for denaturation, 10% acetonitrile (ACN) and 0.5 µg Trypsin-LysC mix (Promega) for 6 h at 37 °C at 450 rpm. The digestion was stopped by addition of 2% trifluoroacetic acid. A desalting of tryptic peptides was performed by the AssayMAP Bravo platform (Agilent Technologies) using C18 cartridges and the "Peptide Clean-Up" program. Samples were eluted with 30 µL of elution buffer (40% ACN, 0.1% formic acid (FA)). Sample were dried at 50 °C for 1 h with a SpeedVac instrument (LabConco) and were suspended in 20 µL of the mobile phase A (0.1% FA in water; Biosolve).

### LC–MS/MS analysis

Tryptic peptide separation was performed using an EvoSep One liquid chromatography system (EvoSep) with a PepSep C18 column (15 cm × 150 µm, 1.5 µm, Bruker Daltonics). A 34 min elution gradient was used corresponding to 30 Sample Per Day (SPD) using mobile phase A (0.1% FA in water; Biosolve) and mobile phase B (0.1% FA in acetontrile; Biosolve). Peptides were analyzed using a trapped ion mobility spectrometry quadrupole time-of-flight mass spectrometer (timsTOF HT, Bruker Daltonics) equipped with a nano-electrospray ion source (Captive spray, Bruker Daltonics) in positive-ion mode. Data were acquired in a Data-Dependent Acquisition (DDA) mode with a TIMS accumulation time of 100 ms and a number of PASEF ramps of 10. The PASEF scan mode was performed in the mass range 10–1700 m/z and ion mobility range 0.75–1.25 1/Ko.

### Proteomic data analysis

Raw files were processed with Skyline-daily software 24.1.1.398 (MacCoss Lab) with following parameters: centroided precursor mass analyzer: MS1 mass accuracy of 10 ppm, centroided product mass analyzer: MS2 mass accuracy of 10 ppm, include all matching scan. The extracted ion chromatograms of selected fragments were manually reviewed and peak picking adjustments were made when needed. The most intense of precursor ion peak area was calculated in Skyline and exported to Excel (Microsoft) for subsequent statistical analysis. To define the tryptic peptide range, all tryptic peptides were aligned on the full-length 2N4R isoform (441 AA).

The peak areas were normalized based on the peak areas of heavy ^15^N-labelled tau standard peptides when possible and corrected for the molar distribution of exon 2, exon 3 and exon 10 heavy ^15^N-labelled tau added. The sum of normalized peak areas is taken for tryptic peptides with a missed cleavage, namely [210–224] and [354–370]. Some oxidations on methionine were detected and considered as artefacts as they may have been induced during sample preparation.

As ^15^N-labelled recombinant tau do not contain PTMs, FragPipe software v22 was used to determine potential tryptic peptides with PTMs of interest such as phosphorylation and ubiquitination. Raw files were processed with MSFragger v4.1 to search for PTMs with serine, threonine and tyrosine phosphorylation and lysine ubiquitination as variable modifications. The database was downloaded directly by the platform from Uniprot Swiss-Prot with protein isoforms, reverse sequences and contaminants. Search parameters were set as follows: trypsin as protease, 2 miss-cleavages, 2 as maximum number of variable modifications, 7–30 amino-acids peptide length range, 20 ppm precursor and fragment mass tolerance. Only peptides with a peptide-spectrum match score above 0.99 were retained.

### Variable domain of the Heavy chain of the Heavy chain only antibodies (VHH) production and biotinylation

To define seed competent tau species enriched in AD EVs, VHH targeting the human tau protein were employed. More specifically, VHH Z70 (tau epitope [305–312], entitled further as VHH-tauRD3) and VHH A5-2 (tau epitope [330–370], entitled further as VHH-tauRD4) were used [44, 45]. A VHH anti-green fluorescent protein (GFP) was used as control. These VHHs used for the IC of tau inside EVs were produced and purified as published in Danis and collaborators and contain a C-terminal cysteine [44]. Briefly, periplasmic extracts of Escherichia coli BL21 (DE3) bacterial cells were retrieved after transformation with various pET22b VHH-cystein and pET22b VHH without C-terminal tag constructs [45]. The VHHs were purified by immobilized-metal affinity chromatography (IMAC, HisTrap HP, 1 mL, Cytiva) followed by SEC (Hiload 16/60, Superdex 75, prep grade, Cytiva) in phosphate buffer (50 mM sodium phosphate buffer [NaPi], pH 6.7, 30 mM NaCl, 2.5 mM EDTA, 1 mM DTT). VHHs were dialyzed against 50 mM Tris pH 8, 50 mM NaCl and cleaved with His-tagged Tobacco Etch virus (TEV) protease. The TEV protease and the cleaved 6-His tag were removed by a second IMAC step, and the VHHs were recovered in the flow-through, concentrated and flash-frozen for further use. A buffer exchange was done to obtain VHHs in PBS (ET330, Euromedex). All recombinant VHHs with a cysteine at the C-terminus were biotinylated with 10 molar excess of maleimide biotin conjugates (A39261, Thermo Fisher Scientific) overnight at 4 °C. The reactions were quenched with 5 mM DTT. Residual biotin conjugates were removed by two consecutive buffer exchanges using a Zeba Spin Desalting column (7 K MWCO, 89882, Thermo Fisher Scientific). The eluate containing the VHH-biotin complex was collected and concentrations were measured using the NanoDrop™ One (Thermo Fisher). Biotinylated VHHs were stored at -80 °C after flash freeze in liquid hydrogen.

### Anti-ubiquitin antibody biotinylation

To perform the single and double IC of ubiquitinated tau, the commercially available mono- and poly-ubiquitinated conjugates recombinant monoclonal antibody (UBCJ2) (ENZ-ABS840, immunoglobulin-1 (IgG1) isotype, ENZO) and a mouse IgG1 isotype control antibody (02-6100, Thermo Fisher) were biotinylated using the EZ link Sulfo-NHS-SS-biotinylation kit (Thermo Fisher, 21445) following the protocol recommendations of the manufacturer. Briefly, 0.4 mg antibody in 0.1 mL filtered PBS was incubated with 10 mM biotin on a wheel at low speed at 4 °C for 2 h in protein low binding eppendorf tubes. After this, the biotinylated antibody was separated from unbound biotin using 0.5 mL Zeba Spin Desalting columns (7 K MWCO, 89882, Thermo Fisher). The eluate containing the antibody-biotin complex was collected and concentrations were measured using the NanoDrop™ One (Thermo Fisher). Biotinylated antibodies were stored at 4 °C.

### EVs tau ID and IC

To assess the seeding capacity of particular tau peptides inside AD-derived EVs, an IC and ID were performed. For this, BD-EVs derived from a pool of three AD patients brain extracts (G, M and N from Table 1) were prepared freshly and sonicated for 30 min in a water bath at 200 W with 30 s intervals (Bioruptor® Sonication System, Diagenode) to which ice was added on regular bases.

In parallel, the IC using the Cytiva streptavidin Mag Sepharose magnetic beads (28985799, Cytiva) was performed as follow. 50 µL streptavidin coated magnetic beads in protein low binding eppendorf tubes were washed in 500 µL of binding buffer (Tris-buffered saline pH 7.5). Next, 60 µg biotinylated VHH (VHH-tauRD3, VHH-tauRD4 or VHH anti-GFP) or 5 µg antibodies (anti-Ubiquitin or anti-IgG1 isotype) in 300 µL binding buffer were added to the beads and incubated for 30 min at room temperature (RT) on a rotor (Grant bio PTR-35) using end over end rotation with intermittent vibrations to ensure successful homogenization. After incubation, unbound VHH or antibody were removed, and two washes were done using 500 µL binding buffer. Then, 300 µL binding buffer containing 1 × 10^10^ AD-EVs (for VHHs-based IC/ID) or 4 × 10^10^ AD-EVs (for antibody-based IC/ID) were added. This mixture was incubated for 1 h at RT on a rotor (Grant bio PTR-35) using end over end rotation with intermittent vibrations to ensure successful homogenization. After this, samples were placed on the magnetic rack. The medium was removed and kept as ID fraction containing all proteins except the tau with the sequence of interest. These 300 µL of ID fraction were concentrated using 3 K amicon to a final volume of 100 µL.

For the elution of VHH-bound tau or antibody-bound tau, 60 µL of 0.1 M glycine–HCl at pH 2.5 were added to the beads for 2 min while tubes were gently shaken. The pH was neutralized by addition of 4 µL of 1 M tris base pH 11 buffer. After mixing and placement on the magnetic rack, the solution containing tau previously captured by the VHH or antibody, called the IC fraction, was collected and the volume was adjusted to 100 µL using PBS.

For the double IC, the IC fraction obtained using anti-ubiquitin was diluted to 300 µL and added on the magnetic beads coupled to the VHH for a second incubation of 1 h. This was followed by collection of the ID fraction and elution of the IC fraction as described above.

The ID and IC using VHH-tauRD3 and anti-ubiquitin antibody of tau from AD brain homogenate were validated by western blot (Supplementary Figs. 3, 4). The enrichment of BD-EVs tau peptides using VHH-tauRD3 or VHH-tauRD4 have also been validated by mass spectrometry analysis as shown in supplementary Fig. 5.

### Cell culture

The stable Tau RD P301S FRET Biosensor cells (ATCC CRL-3275) and HEK293T cells were cultured in Dulbecco’s modified Eagle’s medium (DMEM, Gibco, 13345364) with pyruvate and without HEPES complemented with glutaMax 1X (Gibco-35050061), 10% fetal bovine serum (Gibco, A5256701) and 1% penicillin–streptomycin. The cells were maintained in a humidified incubator with 5% CO_2_. Cell splitting was done twice a week.

### FRET-tau biosensor cell assay

HEK FRET and HEK 293 T cells were plated into a 12-wells plate (150,000 cells per well) 24 h before treatment. Sonicated Tau [244–368], also called K18 fibrils (2 μM, as prepared in Danis and collaborators [44]) were used as a positive control and PBS as a negative control. The ID and IC fractions were lipofected onto the cells. For this, the 100 µL ID or IC fraction was completed with 100 µL of Lipofectamine2000 (Life Technologies, 11668019) diluted at 1:10 in Opti-MEM (Fisher Scientific, 31985062). These transfection mixtures were incubated for 20 min at RT and gently added in the culture medium of the cells. After 72 h, the cells were collected in warm PBS and the Zombi NIR labelling, and fixation protocol were performed as previously described in Leroux and collaborators [32]. The cells were analyzed on the flow cytometer Aria SORP (BD Biosciences; acquisition software FACSDiva v7.0, BD Biosciences) or the Spectral SONY cytometer (SP6800) with the following excitation/emission wavelengths: excitation 405 nm, CFP emission 466 ± 40 nm and FRET YFP 529 ± 30 nm; excitation 488 nm, YFP emission 529 ± 30 nm. The FRET data were quantified using the Kaluza analysis software v2. For VHH IP experiments and double anti-ubiquitin VHH IP experiments three independent experiences each with technical duplicates were performed. For single anti-ubiquitin IP experiments three independent experiences each in singlicate were performed. FACS gating were set to analyze 10,000 YFP + /CFP + double positive cells per replicate in the flow cytometer software and further refined during post-analysis in the Kaluza software, based on the negative control (PBS) included in each experiment. The gating strategy is shown in Supplementary Fig. 6.

### Statistical analyzes

Statistics and graphs were generated using GraphPad Prism 9 software (version 9.1.0). Data were represented as mean ± standard error of mean (SEM). Shapiro–Wilk normality test was used to define normality of each group. Comparison of two independent groups with a normal distribution were done using a t-test and for groups with non-parametric distributions, a Mann–Whitney U test was used. Comparisons of three or more independent groups, with a normal distribution were done using ordinary one-way Analysis Of Variance (ANOVA), while Kruskal–Wallis test was used for non-parametric samples. Statistical testing was done at the two-tailed p-value of 0.05.

## Results

### Numerous tau peptides were detected within BD-EVs from CTRL, AD, PSP and PiD

The tau seeding capacity of BD-EVs from different tauopathies indicates an important heterogeneity [32]. This may be explained by the tau proteoforms and hence, prompt our decision to assess the tau profiles found within the EVs of CTRL, AD, PSP and PiD patients. For this, enzymatic brain dissociation of 4 CTRL, 10 AD, 9 PSP and 4 PiD prefrontal brain samples was followed by SEC for EVs isolation. The EVs quality and characteristics have already been published by Leroux and collaborators [32]. This was complemented with a global proteomics using a Data- Independent Acquisition mode (DIA) which also showed a significant enrichment in EVs-associated proteins compared to proteins known as EVs contaminants (Supplementary Fig. 7).

**Fig. 1:**
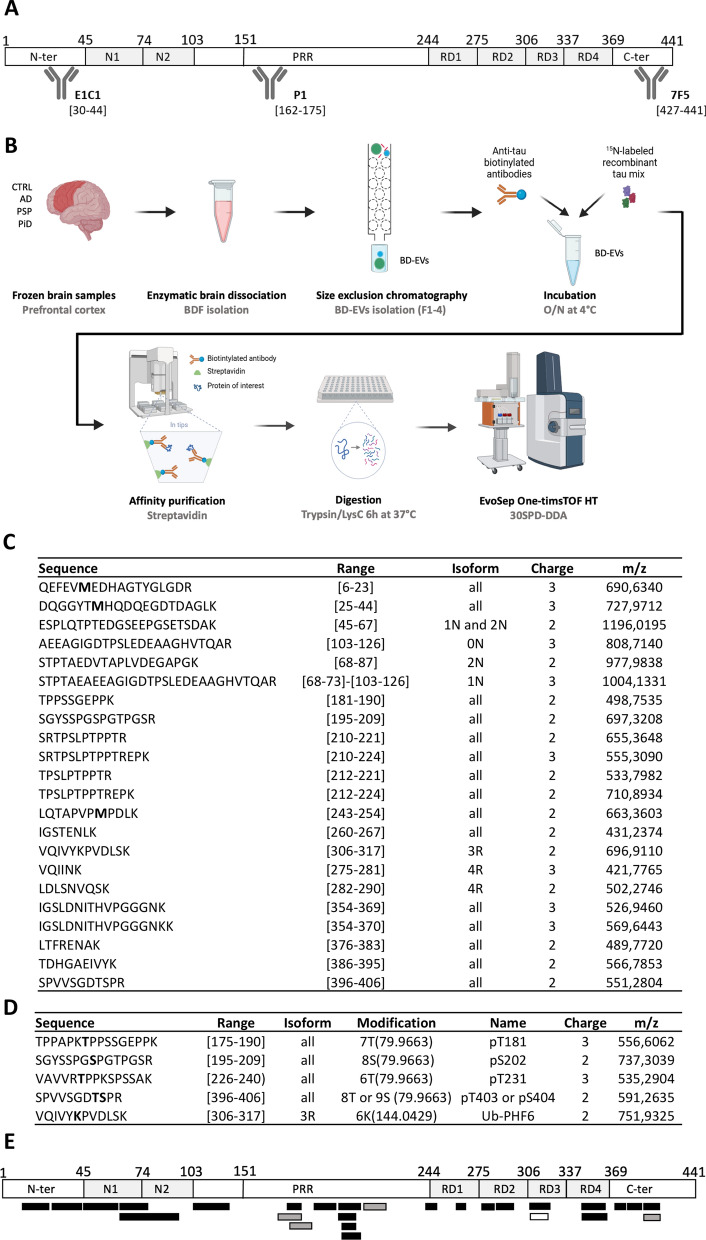
Tau IP-MS of BD-EVs from different tauopathies. **A** Epitopes recognized by antibodies anti-TauE1C1, anti-TauP1 and anti-Tau7F5 used for the tau-enrichment IP-MS aligned on the human 2N4R full-length tau isoform (441 AA). **B** Experimental workflow of the immuno-enriched tau proteomics of BD-EVs. BD-EVs, tau antibodies and recombinant heavy tau isoforms were incubated followed by a streptavidin-based affinity purification and trypsin digestion, prior to the timsTOF HT mass spectrometry analysis. Schematic protocol representations were generated using BioRender ®. **C, D** Summary tables indicating the tryptic tau peptides detected within BD-EVs of CTRL, AD, PSP and PiD. Tryptic peptide range are aligned on the full-length 2N4R isoform (441 AA). **C** Overview listing of tryptic peptides with the amino acid sequence, peptide position range, isoform specificity, charge state and mass-to-charge ratio (m/z). Methionine in bold indicate detection of an oxidation. **D** Displays tryptic peptides carrying a PTM which are phosphorylation (p) or ubiquitination (Ub). Table lists their amino acid sequence, peptide range, isoform specificity, type of modification, PTM name, charge state and m/z. **E** Schematic representation of the sequence coverage of the tryptic peptides detected by mass spectrometry using a scale-based alignment on the full-length tau (441 AA) Tryptic peptides without PTM or an oxidation are indicated in black, tryptic phospho-peptides are indicated in grey and ubiquitinated tryptic peptide are indicated in white

To unravel the tau proteoforms present within BD-EVs by mass spectrometry, a tau-IC was set-up. The combination of three home-made tau antibodies (TauE1C1, TauP1 and Tau7F5) with epitopes across the entire tau sequence were selected to enable the best tau sequence recovery (Fig. 1A, Supplementary Fig. 2C, D). For normalization and hence, comparison among patients and tauopathies, heavy ^15^N-labelled recombinant tau was added along with the antibodies to the BD-EVs. After incubation, a streptavidin-based purification was done followed by trypsin digestion of tau before mass spectrometry analysis (Fig. 1B). In total, 22 peptides of tau, 4 phosphorylated tau peptides and 1 ubiquitinated tau peptide were detected in BD-EVs (Fig. 1C, D). These detected peptides are also represented in a size-scaled alignment on full-length 2N4R tau (Fig. 1E). This tau IP-MS with a mix of three antibodies resulted in a 57.6% tau sequence recovery with the highest (89.8%) recovery in the N-terminal domain (Supplementary Fig. 8). The MTBR and C-terminal domain were found to have a 49.6% and 42.5% sequence recovery, respectively.

**Fig. 2:**
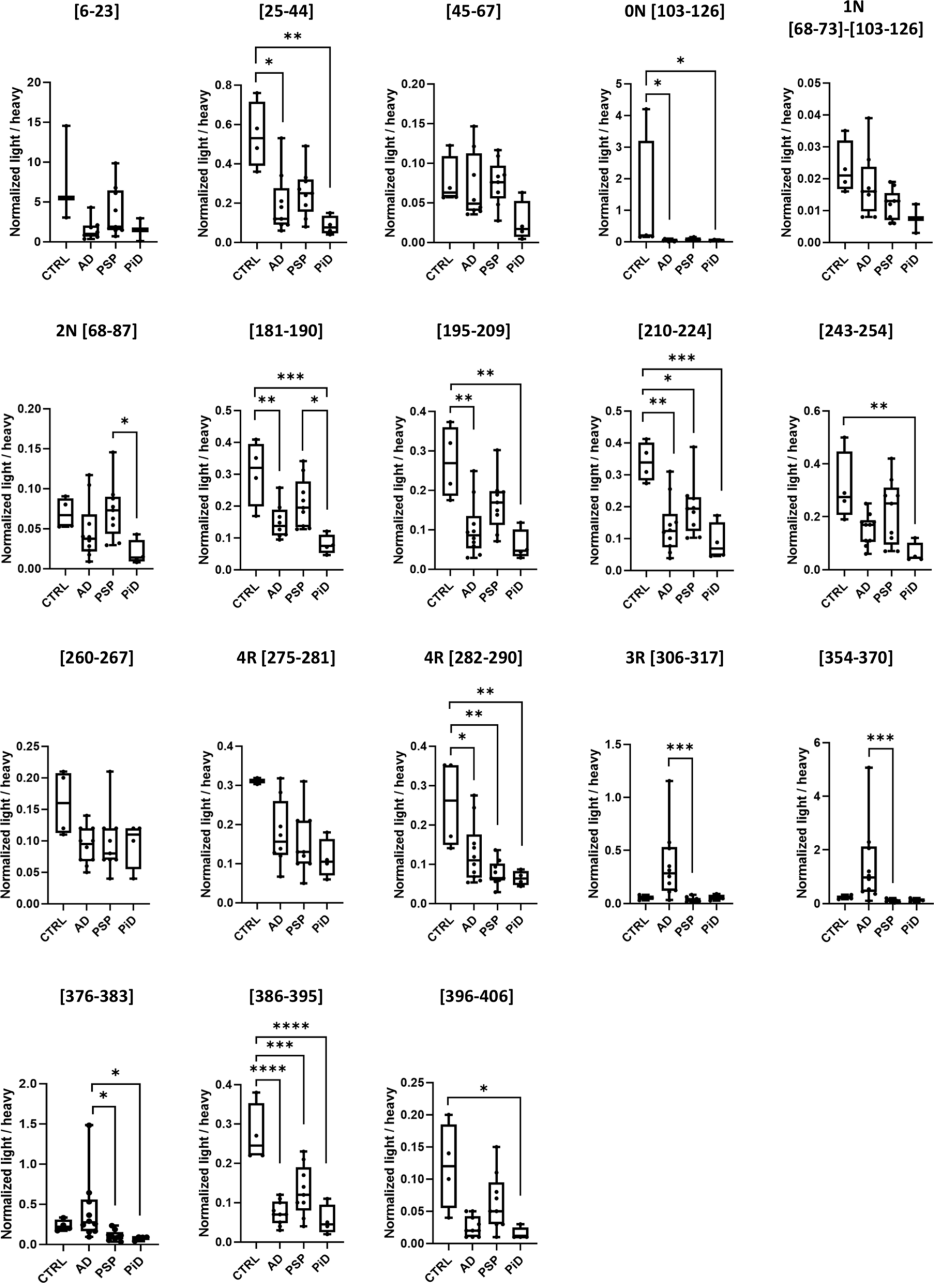
Tau proteoforms in BD-EVs. Boxplots indicating the normalized light-over-heavy ratio. The amino acids range of the tryptic peptides -aligned on 2N4R (441 AA) tau- are indicated between square brackets. Ordinary one-way ANOVA, parametric multiple comparison between all groups for peptides: [181–190], [195–209], [210–224], [260–267], [282–290], [386–395], *p < 0.05, **p < 0.01, ***p < 0.001, ****p < 0.0001. Kruskal–Wallis, non-parametric one-way ANOVA with multiple comparison between all groups for the other tryptic peptides, *p < 0.05, **p < 0.01, ***p < 0.001

We then used the results from the tau-targeted proteomic analysis to investigate the pathophysiology of tau peptides secreted in BD-EVs. For this, a statistical comparison was done between tau peptide levels in CTRL, AD, PSP and PiD BD-EVs (Fig. 2). IP-MS revealed numerous peptides significantly reduced in tauopathies in comparison to CTRL, including [210–224], [282–290], [386-395]. As shown through immunodetection, tau concentration was more elevated in CTRL BD-EVs compared to AD and PiD (Supplementary Fig. 9).

Further, this comparison showed enrichment in PSP compared to PiD for peptides in the N-terminal region ([68–87]) and proline rich region ([181–190]). Interestingly, this comparison also showed that the 3R [306–317], [354–370] and [376–383] peptides are significantly enrichment in AD-derived EVs compared to PSP. This last one is also found significantly enriched in AD compared to PiD, revealing an MTBR enrichment in AD BD-EVs. This comparison highlights heterogeneity in the proteoforms of tau among BD-EVs of different tauopathies.

### PHF6-containing tau in AD BD-EVs is an actor of the EVs-mediated seeding

**Fig. 3:**
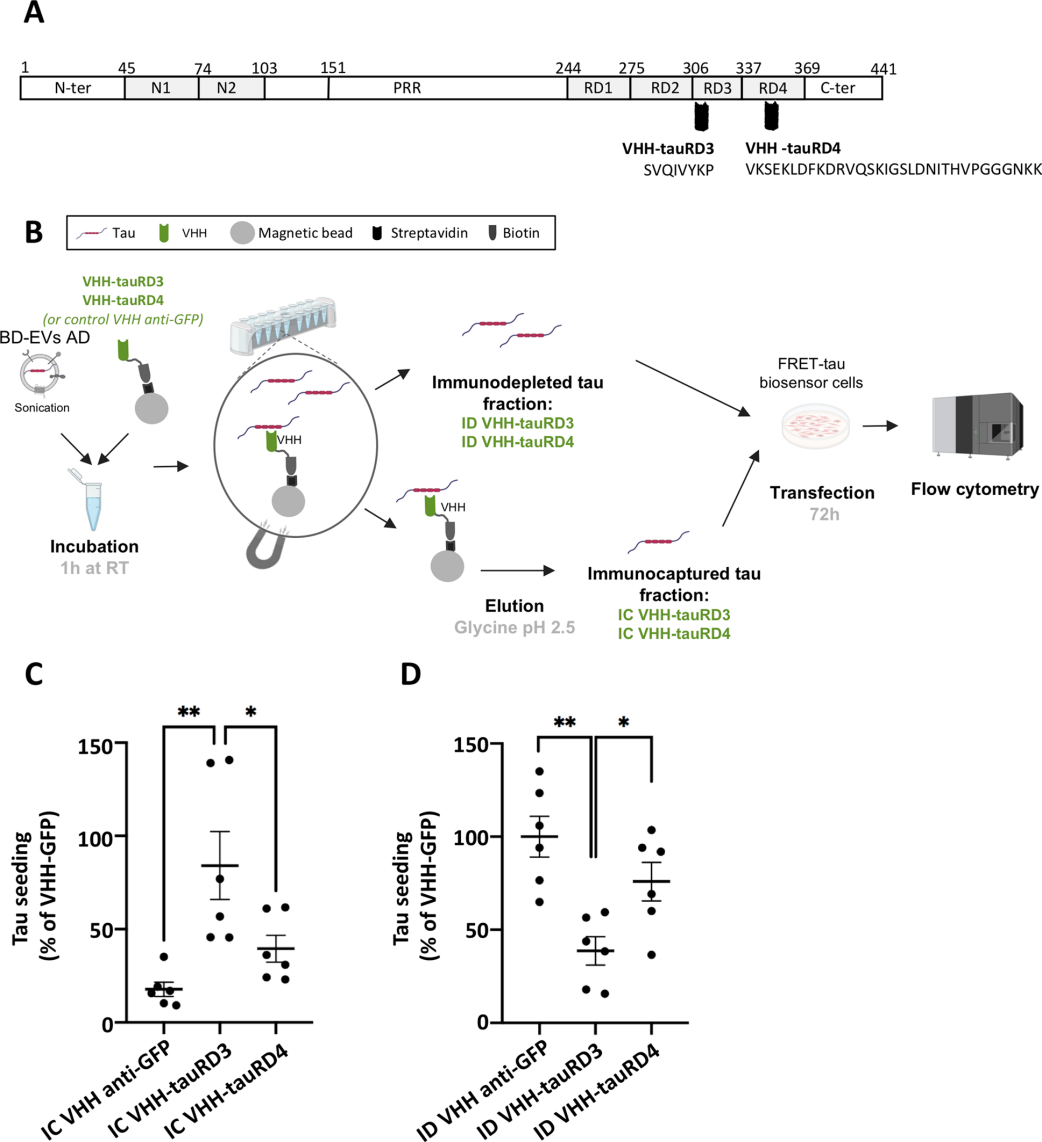
Seeding capacity of AD-enriched tau peptides in BD-EVs. **A** Visual representation of the human 2N4R full-length tau isoform with the VHHs and their epitope sequence used for ID and IC. **B** Experimental workflow of the IC and ID of tau derived from AD BD-EVs. IC and ID fractions were transfected onto the FRET-tau biosensor cell assay and the seeding capacity was quantified by flow cytometry. **C** Dot plot representing the seeding capacity expressed as tau seeding normalized on the ID VHH anti-GFP which was set to 100%. A significant increase is observed after capture of tau containing the VHH-tauRD3epitope and VHH-tauRD4 epitope. Ordinary one-way ANOVA, parametric comparison between all VHH for ID, n = 3 independent experiences (2 technical replicate per experience), *p < 0.05; **p < 0.01. **D** Dot plot representing the seeding capacity expressed as Tau seeding normalized on the ID VHH anti-GFP which was set to 100%. A significant reduction is observed after removal of tau containing the VHH-tauRD3 epitope. Ordinary one-way ANOVA, parametric comparison between all VHH for ID, n = 3 independent experiences (2 technical replicate per experience), *p < 0.05; **p < 0.01

The distinct BD-EVs tau profiles could reflect underlying pathological mechanisms, especially in AD where EVs are implied in tau pathology propagation [31–33]. The peptides of the MTBR have previously been shown enriched in AD sarkosyl-insoluble fractions [46, 47] and recently, Fowler and collaborators found MTBR peptides enriched in AD-EVs as we demonstrate here [31]. Interestingly, one of the AD-enriched peptides, namely 3R [306—317] comprises the PHF6 (VQIVYK) hexapeptide, which is a sequence implicated in tau aggregate formation [34–36]. Hence, we investigated whether these AD-enriched tau sequences: 3R [306–317] and [354–370] transported within EVs explain the high tau seeding capacity we previously observed only in AD patients [32]. To answer this question, immunprecipitations of tau proteins containing these tau sequences in AD BD-EVs were performed using single-domain antibodies: nanobodies®, also known as VHHs. We developed and characterized VHHs directed against different tau epitopes [44, 48–50]. We chose two anti-tau VHHs: one (VHH Z70, entitled further as VHH-tauRD3) targeting the PHF6 sequence of the R3 domain and the other (VHH A5-2, entitled further as VHH-tauRD4) targeting the [354–370] sequence of the R4 domain of tau (Fig. 3A). A VHH targeting GFP was used as a negative control. After sonication, AD BD-EVs were incubated with each VHH coupled to magnetic beads (Fig. 3B). IC and ID fractions were transfected onto HEK FRET-tau biosensor cells. The FRET-positive percentage of IC fractions was normalized against VHH anti-GFP ID (theoretically containing all seeding species) set at 100% (Fig. 3C, D). Our results show a significant increase in tau seeding for PHF6-containing proteins (IC VHH-tauRD3) and no significant increase in tau seeding for [354–370] epitope-containing proteins (IC VHH-tauRD4) compared to VHH anti-GFP. The IC VHH-tauRD3 therefore confirms the best enrichment of pro-nucleating species.

Similarly, ID fractions containing tau proteins unbound to VHH-tauRD3 or VHH-tauRD4 were collected and transfected onto HEK FRET-tau biosensor cells. As for IC, the ID fraction with VHH anti-GFP containing all tau seeds was fixed at 100% and used for normalization of the other ID fractions (Fig. 3D). Our results show that the ID fraction of tau proteins lacking the PHF6 sequence (ID VHH-tauRD3) in AD BD-EVs has a significantly decreased tau seeding capacity of 61% compared to VHH anti-GFP (Fig. 3D). In contrast, the remaining tau proteins in the VHH-tauRD4 immunodepleted fraction retained tau seeding capacity. Altogether, these results confirm that among the VHH-targeted tau proteins enriched in AD BD-EVs, tau proteins containing the PHF6 sequence are the most involved in EV-mediated tau seeding.

### Ubiquitinated tau peptides detected inside BD-EVs of AD

**Fig. 4:**
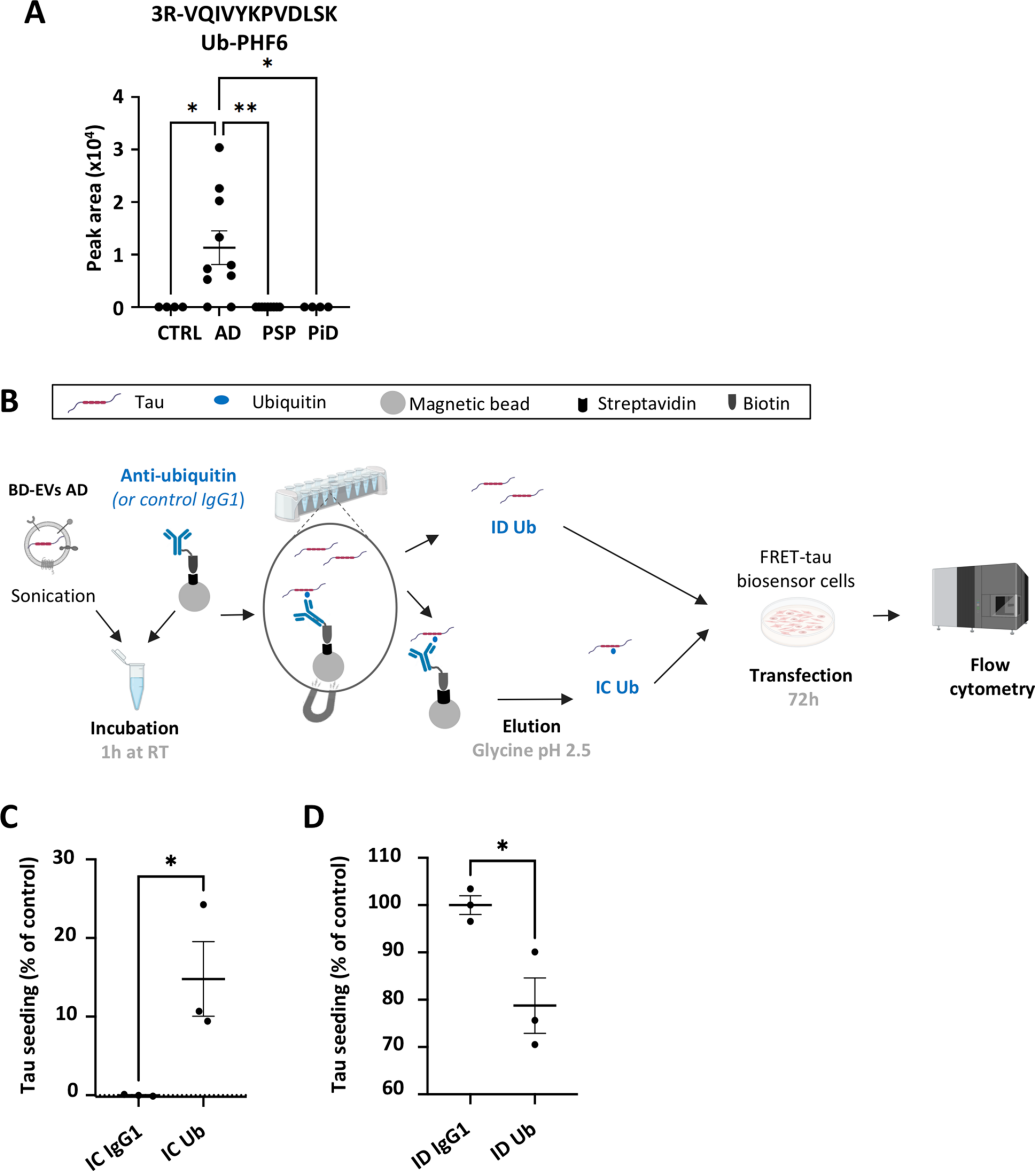
Seeding capacity of AD-enriched ubiquitinated tau peptides in BD-EVs. **A** Boxplot indicating the detection of Ub-PHF6 in BD-EVs from CTRL, AD, PSP and PiD. Kruskal–Wallis, non-parametric one-way ANOVA with comparison between all conditions, *p < 0.05, **p < 0.01. **B** Experimental workflow of the single ID and IC of ubiquitinated tau derived from AD BD-EVs using an IgG1 anti-ubiquitin or an IgG1 isotype control. **C, D** Dot plots representing the seeding capacity of the single IC fractions (**C**) and ID fractions (**D**) expressed as Tau seeding normalized to the ID anti-IgG1 isotype control which was set to 100%. Capture of ubiquitinated tau species (IC Ub) and depletion of ubiquitinated tau species (ID Ub) show respectively a significant increase and decrease compared to the isotype control. Unpaired t-test, parametric comparison between IgG1 and anti-ubiquitin, n = 3 independent experiences (in singlicate), *p < 0.05

Given the significant recovery of tau peptides, we further analyzed these peptides for disease-relevant PTMs, which play a pivotal role in tau pathology. Phosphorylation is the best-studied tau PTM [6]. Here, a total of four phospho-peptides (pT181, pS202, pT231 and pT403/pT404) were detected inside BD-EVs among tauopathies (Supplementary Fig. 10), with the majority located in the PRR (Fig. 1E). Of these, pT181 was shown significantly enriched in BD-EVs of CTRL compared to PiD, and pT403/pS404 was found enriched in BD-EVs of CTRL compared to AD. Further, no tau acetylation was detected. Nevertheless, tau is a lysine-rich protein and is therefore susceptible to ubiquitination, which regulate tau clearance [8, 51]. Our tau-enriched IP-MS did reveal ubiquitination on the 3R [306—317] peptide (Ub-PHF6) only in AD-EVs (Fig. 4A). Interestingly, this is located on the PHF6 sequence that we previously shown to have a high implication in EVs-mediated seeding (Fig. 3C). To investigate whether ubiquitinated forms of tau could have a pro-seeding capacity, we performed anti-ubiquitin immunoprecipitation on AD BD-EVs and analyzed IC and ID fractions (Fig. 4B). An anti-IgG1 was used as an isotype control. The ID and IC fractions were transfected in the FRET-tau biosensor cell assay. The ID fraction with the IgG1 isotype, theoretically containing all AD BD-EVs tau proteins, was set to 100% for normalization of all ID and IC conditions (Fig. 4C, D). As expected, the IC IgG1 (negative control) did not contain any pro-seeding tau species. In contrast, the IC Ub fraction enriched in ubiquitinated tau species showed some tau seeding capacity, although this remained relatively low around 15% of the total BD-EV tau seeding capacity (Fig. 4C). The moderate immunocapture efficiencies obtained with anti-ubiquitin antibody (Supplementary Fig. 4) could in part explain the significant but relatively low seeding capacity of ubiquitinated tau forms. For the ID fractions, fraction ID IgG1 includes all pro-nucleating tau species, while fraction ID Ub contains less ubiquitinated tau. We observe a significant decrease in seeding capacity after removal of ubiquitinated forms of tau (Fig. 4D).

**Fig. 5:**
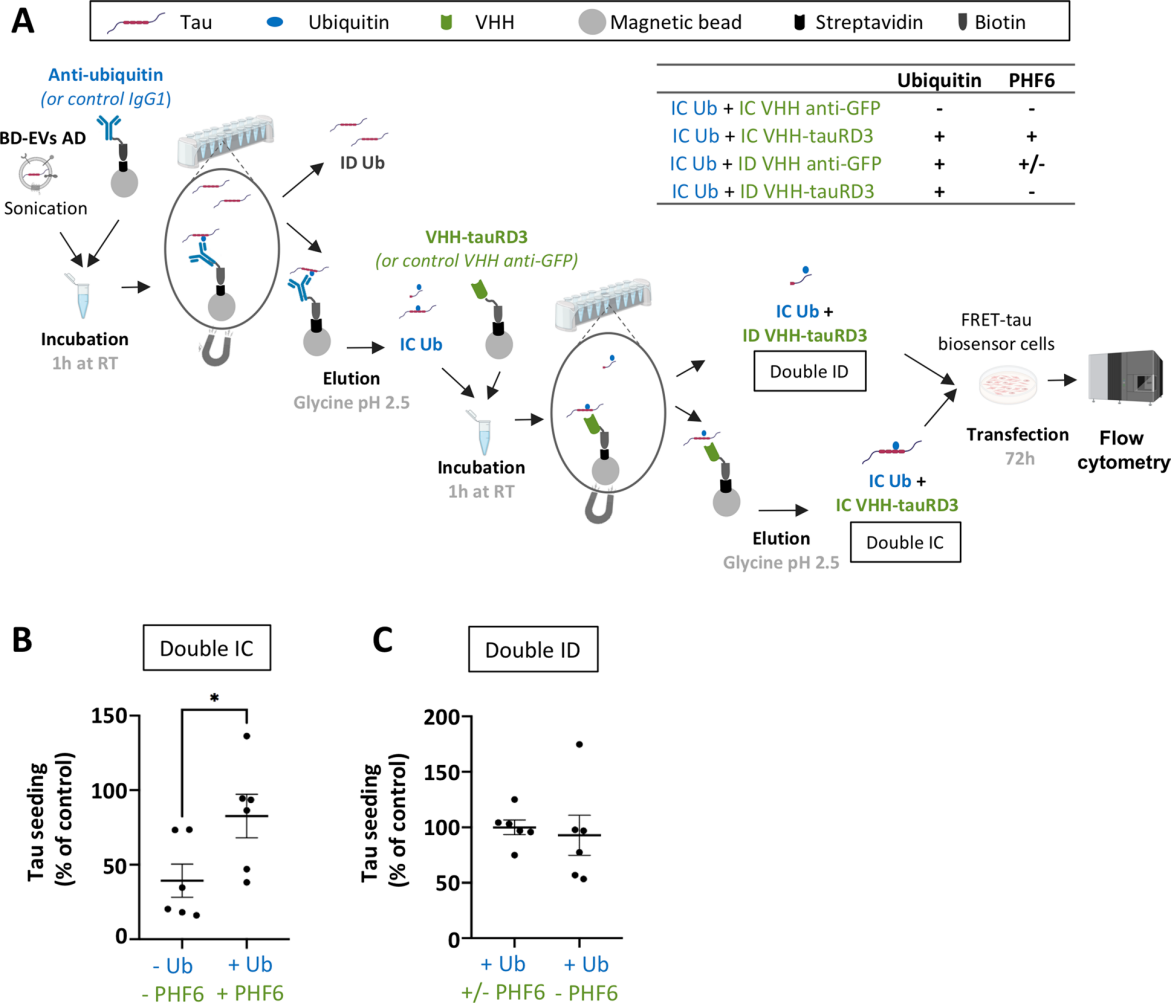
Seeding capacity of AD-enriched ubiquitinated tau peptides containing the PHF6 in BD-EVs. **A** Experimental workflow of the double immunoprecipitation. First, ubiquitinated tau were captured using an anti-ubiquitin and underwent a second immunoprecipitation (VHH-tauRD3) to enrich in tau containing both an ubiquitin and the PHF6 sequence. VHH anti-GFP was used as isotype control. **B**, **C** Dot plots representing the seeding capacity of double IC (Ub and VHH) (**B**) and double ID (Ub and VHH) (**C**) fractions expressed as Tau seeding normalized to the IC Ub + ID anti-GFP fraction containing all ubiquitinated tau with and without PHF6 sequence (positive control). Double IC; Mann–Whitney test, non-parametric comparison, n = 3 independent experiences (2 technical replicates per experience), *p < 0.05) and double ID; Unpaired t-test, n = 3 independent experiences (2 technical replicate per experiences)

Now, among ubiquitinated tau, we aim to study the seeding capacity of Ub-PHF6 tau, which we found enriched by mass spectrometry (Fig. 4A). For this, we immunocaptured ubiquitinated-tau proteins (anti-ubiquitin antibody) which then underwent a second immunoprecipitation to select proteins containing the PHF6 sequence using VHH-tauRD3 (Fig. 5A). VHH anti-GFP was used as a control. The IC and ID fractions from the double immunoprecipitation were transfected onto the FRET-tau biosensor cell assay to analyze their ability to induce tau seeding. The IC Ub + ID anti-GFP fraction, containing all ubiquitinated tau (with and without PHF6 sequence) was set to 100% for normalization. The results indicate that tau species containing both an ubiquitination and the PHF6 sequence (Fig. 5B: Ub + , PHF6 +) in AD-derived EVs have a higher seeding capacity than the negative controls (Fig. 5B: Ub-, PHF6-). Selective removal of ubiquitinated tau proteins containing the PHF6 (Fig. 5C: Ub + , PHF6-) did not result in a significant reduction in seeding capacity. This suggests that ubiquitinated forms of PHF6 tau species may induce pro-seeding activity but it remains limited.

## Discussion

Tauopathies, although all characterized by the presence of tau lesions within the brain, are heterogeneous based on their clinical symptoms, tau filament cryo-electron microscopy (EM) conformation and isoform, affected cell type, spatio-temporal staging and many more [15]. Recently, it was demonstrated that BD-EVs -mediators of the intercellular transfer- isolated from the prefrontal cortex- have a seeding capacity across all three tauopathies (AD, PSP and PiD). Although with varying efficiencies, notably with AD EVs having the highest seeding capacity [32]. This could be related to the nature of tau species transported within BD-EVs. At this day, only one study has identified the tau proteoforms of AD BD-EVs [31]. In this article, we went further and identified the tau profiles present within BD-EVs from AD, PSP, PiD and CTRL. For this, we performed an IC of tau coupled to mass spectrometry analysis (tau IP-MS), revealing detection of 22 tryptic tau peptides in all conditions and without phosphorylation or ubiquitination, with a total sequence recovery of 57.6%. Although the tau recovery was well successful, it does not exclude the loss of a few peptides because of their low abundance or ionization capacity.

This proteomic analysis showed a higher abundance for numerous tau tryptic peptides for CTRL (Fig. 2), confirming the elevated Simoa quantification of total tau in CTRL BD-EVs (Supplementary Fig. 9). We then wondered whether the tau content could be altered during tau pathology. Since there was no significant difference in total tau levels quantified by Simoa among AD, PSP and PiD BD-EVs (Supplementary Fig. 9), the tau IP-MS peptide abundance allowed to compare if particular tau tryptic peptides in EVs could be tauopathy specific or enriched. We found several tau peptides enriched in PSP or AD which open prospects in accessible biological fluids such as CSF or plasma for differential diagnosis of tauopathies. Further, studying tauopathies, we assessed if the dominant isoform found within tau filaments of tauopathies is reflected in the isoform distribution inside EVs (Supplementary Fig. 11). Whereas a correspondence was found for the 4R-tauopathy PSP, we did not find more 3R isoforms in BD-EVs derived from PiD. Previous proteomic studies indicated that isoform distribution of sarkosyl-insoluble fractions correlate with the dominant isoform in filaments of tauopathies [47, 52]. Hence, our findings suggest that BD-EVs from tauopathies do not necessarily mirror the isoform composition of the aggregates. Ruan and collaborators studied the BD-EVs of AD patients and found enrichment of oligomeric tau compared to control BD-EVs [33]. Nevertheless, some paired helical filaments (especially in very large-sized EVs) have been detected in BD-EVs of AD [31, 33], and therefore it is of interest to map the relative proportion of oligomeric tau and filaments within BD-EVs and to assess their contribution to tau seeding. To fully map these different tau structural assemblies (truncated tau, oligomeric tau, filaments…) within BD-EVs from tauopathies, cryo-EM, high-performance liquid chromatography (HPLC)-coupled SEC or top-down proteomics is required in the future.

The tau IP-MS showed peptides of the MTBR RD3 and RD4 significantly enriched in BD-EVs of AD compared to CTRL, as equally observed by Fowler and collaborators [31]. MTBR has also been described in the cerebro-spinal fluid and in sarkosyl-soluble and sarkosyl-insoluble fraction of AD patients [47, 53]. Enriched MTBR peptides in AD-EVs may explain why these vesicles are more seed competent than EVs from other tauopathies [32]. Therefore, IC and ID were performed to study the seeding capacity of tau containing one of these two AD enriched tau sequences using two different VHHs: VHH-tauRD3 (VHH Z70) and VHH-tauRD4 (VHH A5-2). The ID VHH-tauRD4 and VHH-tauRD3 showed 27.8% and 50.8% reduction of the targeted peptides, respectively. IC VHH-tauRD4 had a capture efficiency of 1.4%, and VHH-tauRD3 of 29.6% measured by mass spectrometry (Supplementary Fig. 5). The equilibrium dissociation constant (K_D_) of VHH-tauRD4 (K_D_ = 576 nM) was approximately two times higher than VHH-tauRD3 (K_D_ = 246 nM) meaning more tau dissociation occurs for VHH-tauRD4. Also, the Koff values are different (koff = 56.3 × 10^–3^ s − 1 for VHH-tauRD4 and 3.53 × 10^–3^ s − 1 for VHH-tauRD3 (Supplementary Fig. 12) with VHH-tauRD3 Koff 16 times lower than VHH-tauRD4 [45]. Together these VHH parameters could explain these results. By transfecting ID and IC VHH-tauRD3 fractions on the FRET-tau biosensor cell assay, we demonstrated that the tau proteins in AD-EVs containing the PHF6 hexapeptide have a high seeding capacity (Fig. 3C, D). The low capture efficiency combined with the seeding capacity of tau containing the IGSLDNITHVPGGGNKK [354–370] sequence suggests that this tau specie might also be implicated in EV-mediated seeding although we cannot exclude that it also contains the PHF6 sequence (Fig. 3C, D). Multiple research teams have demonstrated that the PHF6 (VQIVYK) hexapeptide is an active core for the templated misfolding [34, 35, 54–56]. Recently, Lövestam and collaborators visualized the implication the PHF6 as forming the first intermediary amyloid filaments, acting as the primary nucleation of recombinant tau by cryo-EM [36]. Our data clearly demonstrated that, in addition to its pro-aggregative role inside the neuron, the PHF6 motif is also a seed competent peptide implicated in human EVs-mediated pathology progression. In the future, it would be of interest to map at which Braak stage, the enriched abundance of PHF6-containing tau in the BD-EVs occurs to understand the kinetics of propagation via EVs.

The tau protein can undergo numerous PTMs, which has driven research to map disease-specific PTMs. Studying the sites of tau phosphorylation, we detected only four phospho-peptides and found a decrease in pT403/pS404 tau in AD-EVs compared to CTRL (Supplementary Fig. 10). The low abundance of phosphorylated tau in BD-EVs may result either from reduced secretion of phosphorylated tau through EVs or from post-mortem dephosphorylation driven by phosphatase activity within BD-EVs [57]. However, other research groups have shown in AD-EVs the presence or enrichment of pS404 tau while using the conformational dependent PHF-1 antibody for western blot [31, 33]. The observed differences are likely technique dependent as mass spectrometry allows detection of tau phospho-peptides in a conformation independent manner allowing detection of numerous phosphorylations in CTRL BD-EVs. The four detected phospho-peptides are known to be phosphorylated in both physiological conditions and in AD [58]. Interestingly, they are also detected in BD-EVs of PSP and PiD. Our results suggest a basal transport of phospho-tau species within EVs, although the majority of tau transported within EVs is non-phosphorylated.

Based on our tau IP-MS, we detected ubiquitination on the PHF6-containing tryptic peptide (3R-specific) unique to AD-EVs. This Ub-PHF6 has been previously described to be enriched in AD patients brain lysate (soluble and insoluble fractions) [52, 59], but this is the first demonstration that it is also present within AD BD-EVs. As we showed a crucial role of the PHF6-containing tau species inside EVs for tau seeding, we assessed if Ub-PHF6 tau species from EVs present a seeding capacity. First, we enriched in ubiquitinated tau species from EVs, which revealed a capacity to induce tau nucleation. We then aim to specifically study Ub-PHF6 tau. Despite the low efficiency of Ub-PHF6 capture (Supplementary Fig. 3–5), these species from AD BD-EVs proved to be seed competent. Previously, research has shown that ubiquitination can enhance tau aggregation assembly, and it was hypothesized to be related to the neutralization of the MTBR positive charge [60–63]. The occurrence of ubiquitinated tau inside EVs, drives the hypothesis that affected cells have saturated ubiquitin–proteasome degradation systems and hence try to discard this pathological tau via encapsulation in EVs for degradation in other brain cells (incl. microglia or healthy neurons) [64, 65]. Another explanation might be that some PTMs affect the addressing of protein to degradation systems.

## Conclusions

Our observations highlight that the tau profile within BD-EVs is different among tauopathies, with enrichment of PHF6-tryptic peptide in AD BD-EVs. We demonstrate a crucial role of the 3R-specific PHF6-containing tau, in EVs-mediated tau seeding of AD. This insight advances our understanding of AD mechanisms by showing a sorting process that favors PHF6 seeding-competent tau into EVs. Hence, the cellular machinery possibly contributes to loading toxic tau conformers into EVs driving to disease progression.

## Supplementary Information

Additional file 1.

## Data Availability

The datasets used and/or analyzed during the current study are available from the corresponding author on reasonable request. Proteomics data obtained by mass spectrometry are deposited on PanoramaWeb (https://panoramaweb.org/Qr5Ik5.url) and they are also available in the PRIDE repository (ProteomeXchange) with the identifier PXD067593.

## Abbreviations

AD: Alzheimer’s disease
BD-EVs: Brain-derived EVs
DDA: Data-dependent acquisition mode
DIA: Data-independent acquisition mode
EM: Electron microscopy
EVs: Extracellular vesicles
FRET: Fluorescence resonance energy transfer
FA: Formic acid
GFP: Green fluorescent protein
HPLC: High- performance liquid chromatography
IMAC: Immobilized-metal affinity chromatography
IgG1: Immunoglobulin-1
IC: Immunocapture
ID: Immunodepletion
MTBR: Microtubule-binding region
NTA: Nanoparticle tracking analysis
NHS: N-hydroxysuccimide
CTRL: Non-demented human control
NMR: Nuclear magnetic resonance
ANOVA: Ordinary one-way Analysis Of Variance
PiD: Pick’s disease
PTMs: Post-translational modifications
PSP: Progressive Supranuclear Palsy
PRR: Proline rich region
RD: Repeat domain
RT: Room temperature
SEM: Standard error of mean
SEC: Size-exclusion chromatography
Simoa: Single molecule assay
tau IP-MS: Tau immunoprecipitation followed by mass spectrometry analysis
TEV: Tobacco Etch virus
Ub-PHF6: Ubiquitinated PHF6
UPS: Unconventional protein secretion
VHH: Variable domain of the Heavy chain of the Heavy chain only antibodies
