# Supplementary material for: Tau profiling of brain extracellular vesicles reveals PHF6 peptide as core for pathological tau seeding in Alzheimer's disease
Source: J Biomed Sci. 2026 Jun 15;33:62. doi: 10.1186/s12929-026-01250-1 (PMC13267276; doi:10.1186/s12929-026-01250-1)
Supplement: Supplementary file 1 — Additional file 1. [file 12929_2026_1250_MOESM1_ESM.pdf]

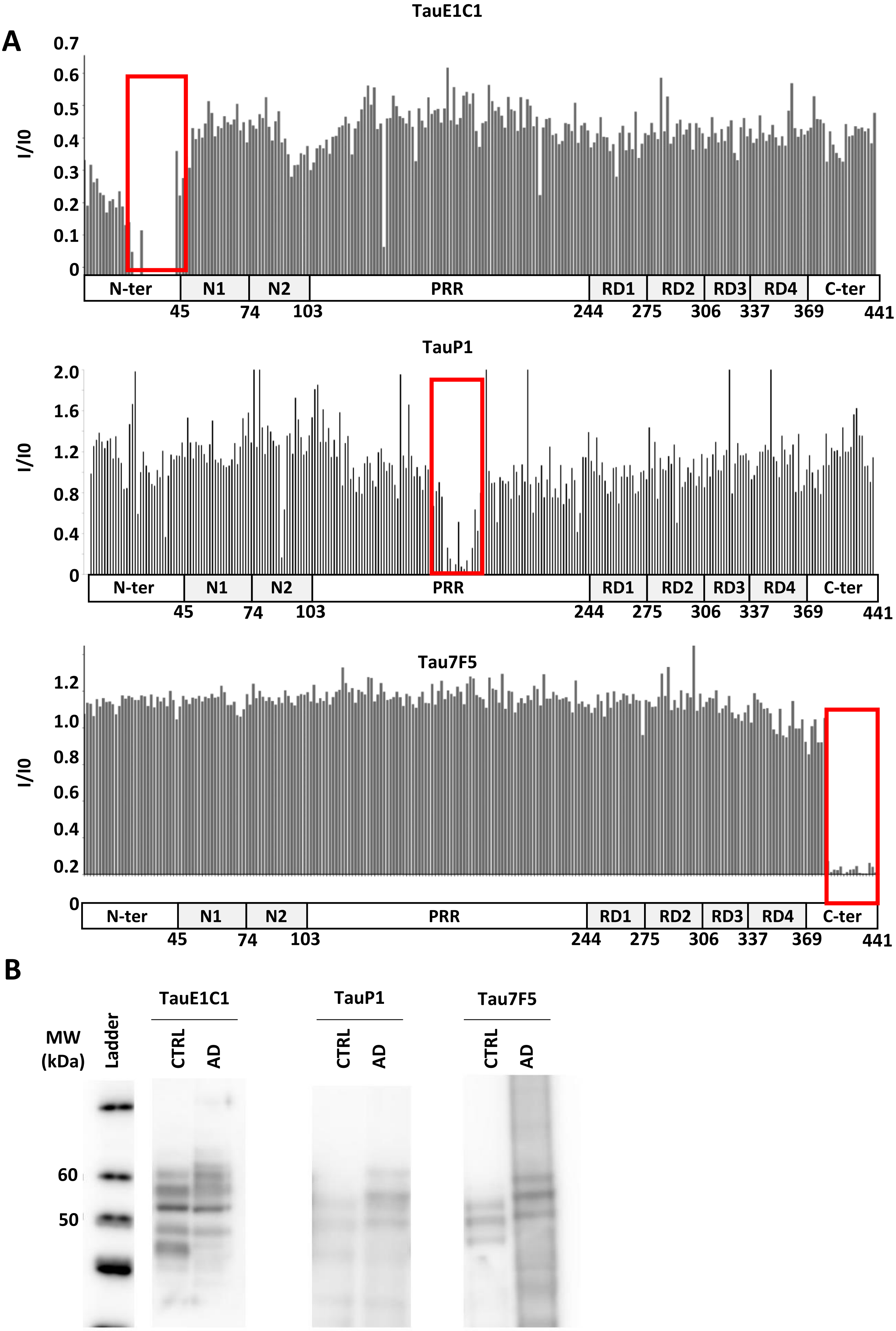

Supplementary figure 1: **Anti-tau antibodies.** To enrich in tau prior to mass spectrometry, a mix of three anti-tau antibodies were used. This includes the home-made anti-Tau Exon1 (TauE1C1), anti-TauPRR (TauP1) and anti-TauCter (Tau7F5). **(A)** Comparison of NMR spectrums for the three antibodies expressed as normalized NMR intensity (I/I0) along the full-length tau sequence. I0 corresponds to the ^{1}H resonance intensity of free tau in solution, and I represents the resonance intensity of tau mixed with equimolar amounts anti-TauE1C1, anti-TauP1 or anti-Tau7F5. This results show that anti-TauE1C1 recognizes N-terminal tau amino acid sequences [30-44], TauP1 recognizes tau amino acid sequence [162-175] in the proline rich region and Tau7F5 recognizes C-terminal tau amino acid sequence [427-441] as antibody epitope (indicated in red). **(B)** Using western blotting the antibodies were validate for successful detection of tau from non-demented (CTRL) and Alzheimer's Disease (AD) brain lysate.

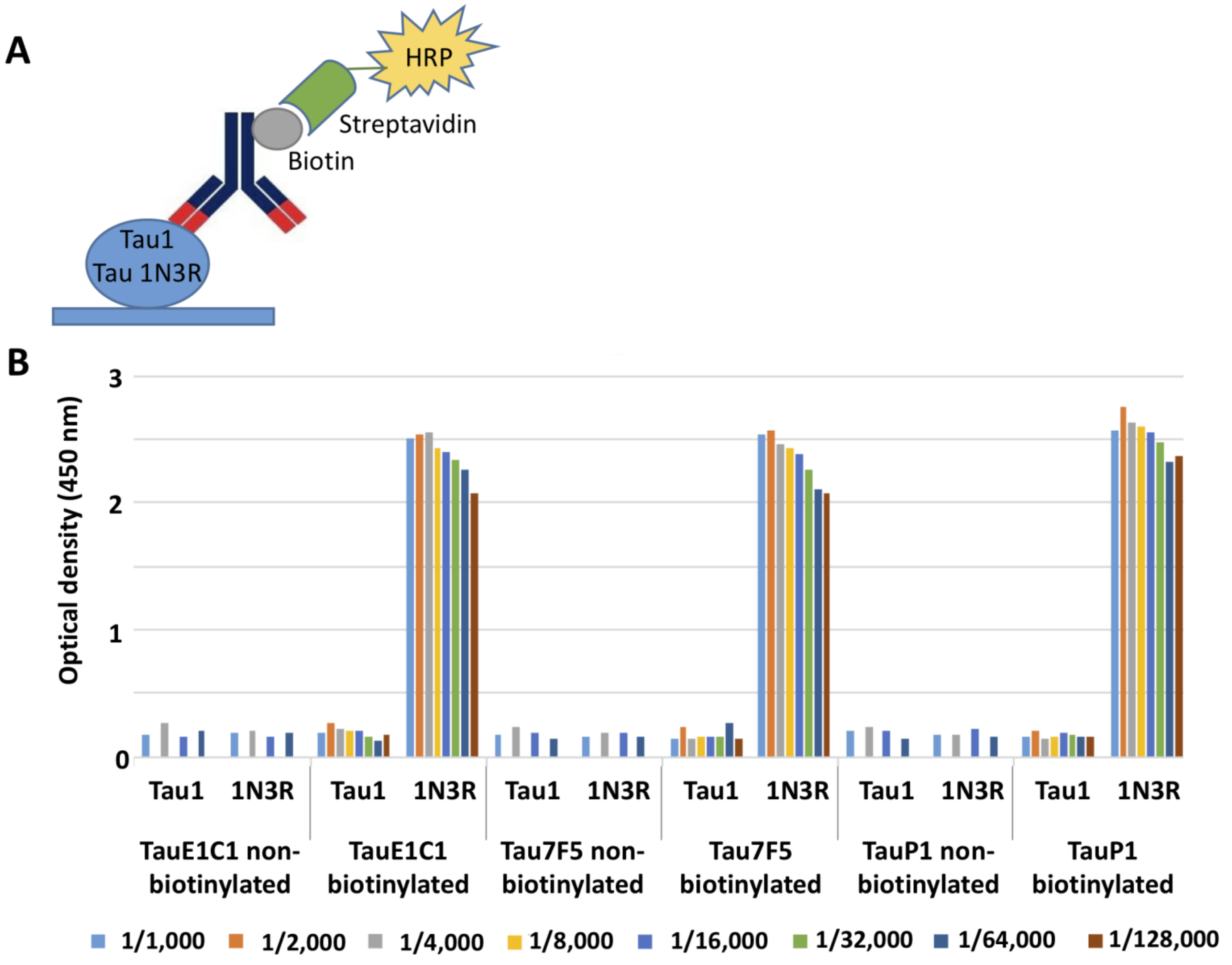

C

IP-MS	Number of detected tau peptides
Anti-TauP1 alone	12
Anti-TauE1C1 alone	11
Anti-Tau7F5 alone	13
Combination anti-TauE1C1, anti-TauP1 and anti-Tau7F5	15

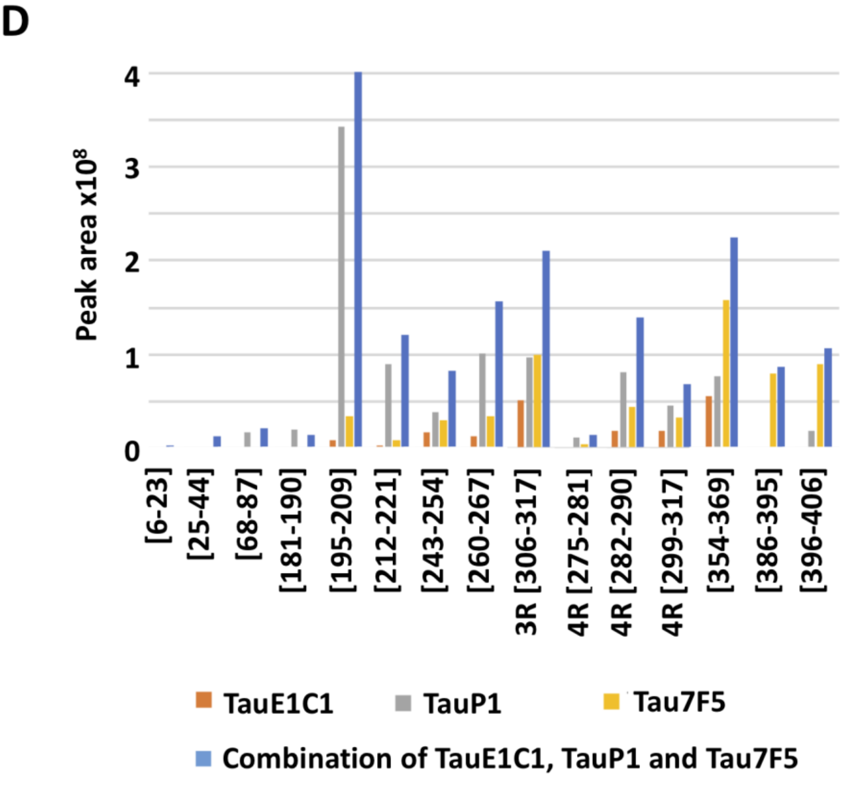

Supplementary figure 2: **Immunocapture for tau-enrichment prior to mass spectrometry. (A)** Schematic representation of the home-made ELISA performed to validate the biotinylation of the anti-tau antibodies (TauE1C1, Tau7F5 and TauP1). Recombinant Tau1 and 1N3R tau were used as coating, where Tau1 [1-21] has a sequence range not present within the antibody epitopes and hence was used as negative control. **(B)** Histogram indicating successful biotinylation of TauE1C1, Tau7F5 and TauP1. **(C)** Table indicating the number of detected tau peptides from AD-derived EVs using either TauP1 alone, TauE1C1 alone, Tau7F5 alone or a combination of the three antibodies. **(D)** Histogram indicating the different tau peptides listed in table C. The data presented were generated by LC-MS/MS analysis with the nanoElute (Bruker) coupled to the Q-tof system (Impact IITM; Bruker). More tau peptides with higher peak areas were recovered using the combination of TauE1C1, TauP1 and Tau7F5.

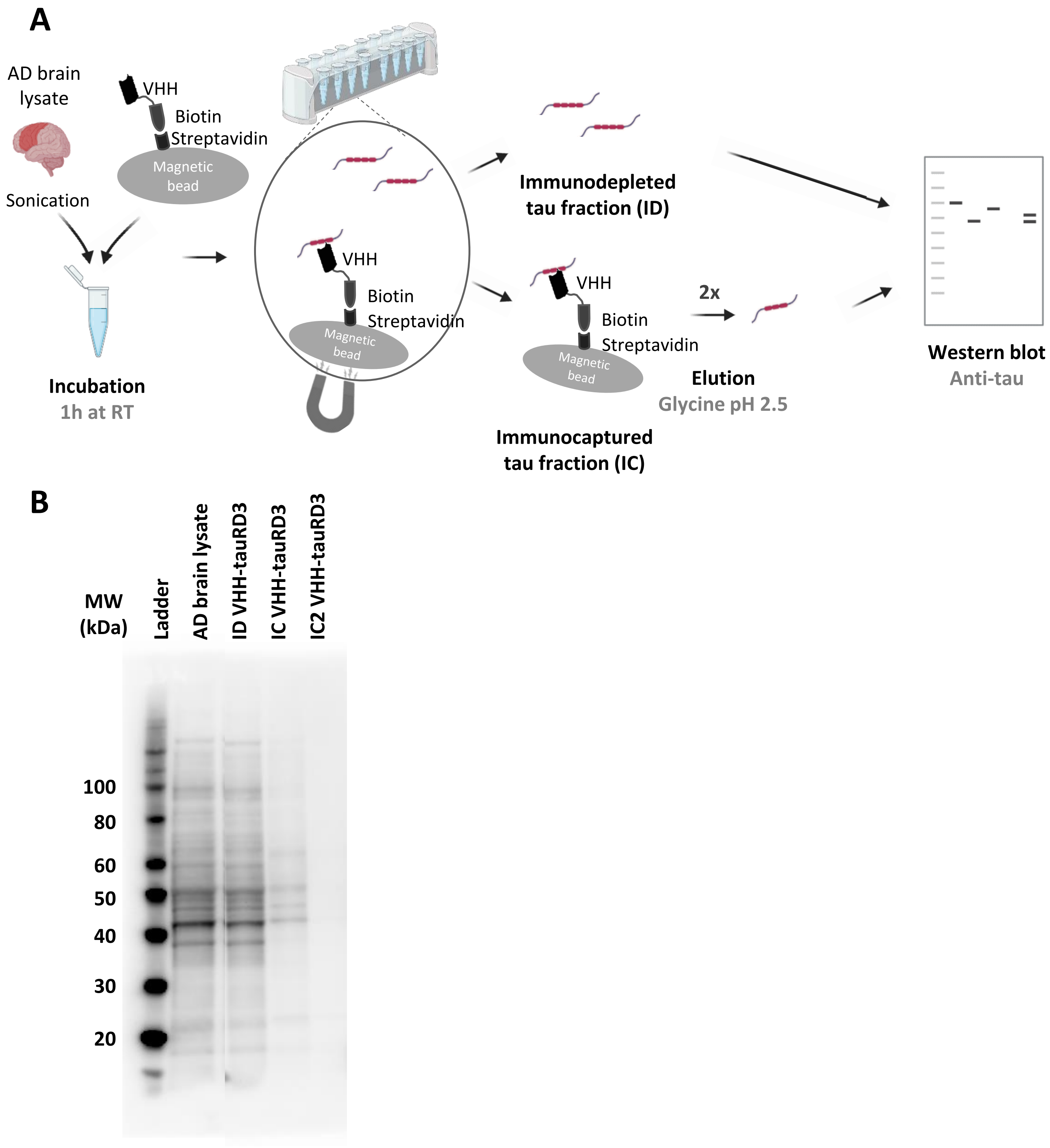

Supplementary figure 3: **Western blot validation of the immunocapture and immunodepletion protocol using VHHs. (A)** Schematic overview of the immunodepletion and immunocapture workflow using biotinylated VHH-tauRD3. This was done on Alzheimer's Disease brain lysate (40 µg). **(B)** Western blot of ID, IC and IC2 fraction using home-made anti-tau antibody against the MTBR [244-369] (4A10, 1/1000) and Trueblot ultra anti-mouse IgG-HRP (18-8517-33, Rockland) reveals PHF6 containing tau in the IC fraction and not in the IC2 fraction.

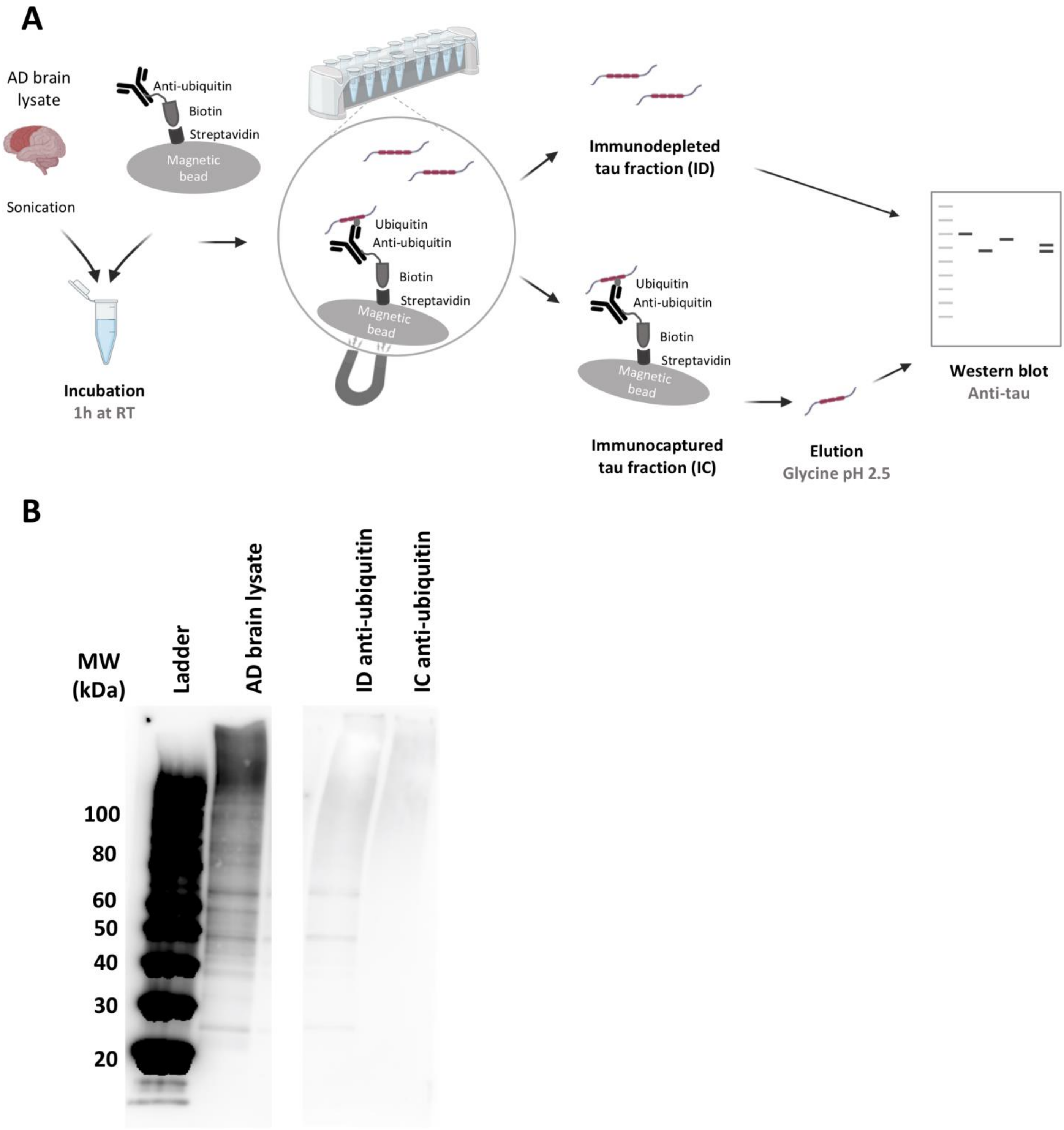

Supplementary figure 4: **Western blot validation of the immunocapture and immunodepletion protocol using anti-ubiquitin antibody. (A)** Schematic overview of the immunodepletion and immunocapture workflow using biotinylated anti-ubiquitin (ENZ-ABS840; 2µg). This was done on Alzheimer's Disease brain lysate (40 µg). **(B)** Western blot of the ID and IC fraction using a home-made anti-tau antibody against the C-terminal region (993S1, 1/4000) and goat anti-rabbit IgG(H+L) peroxidase (PI-1000, vector laboratories, 1/25 000) reveals the capture of tau in the IC fraction.

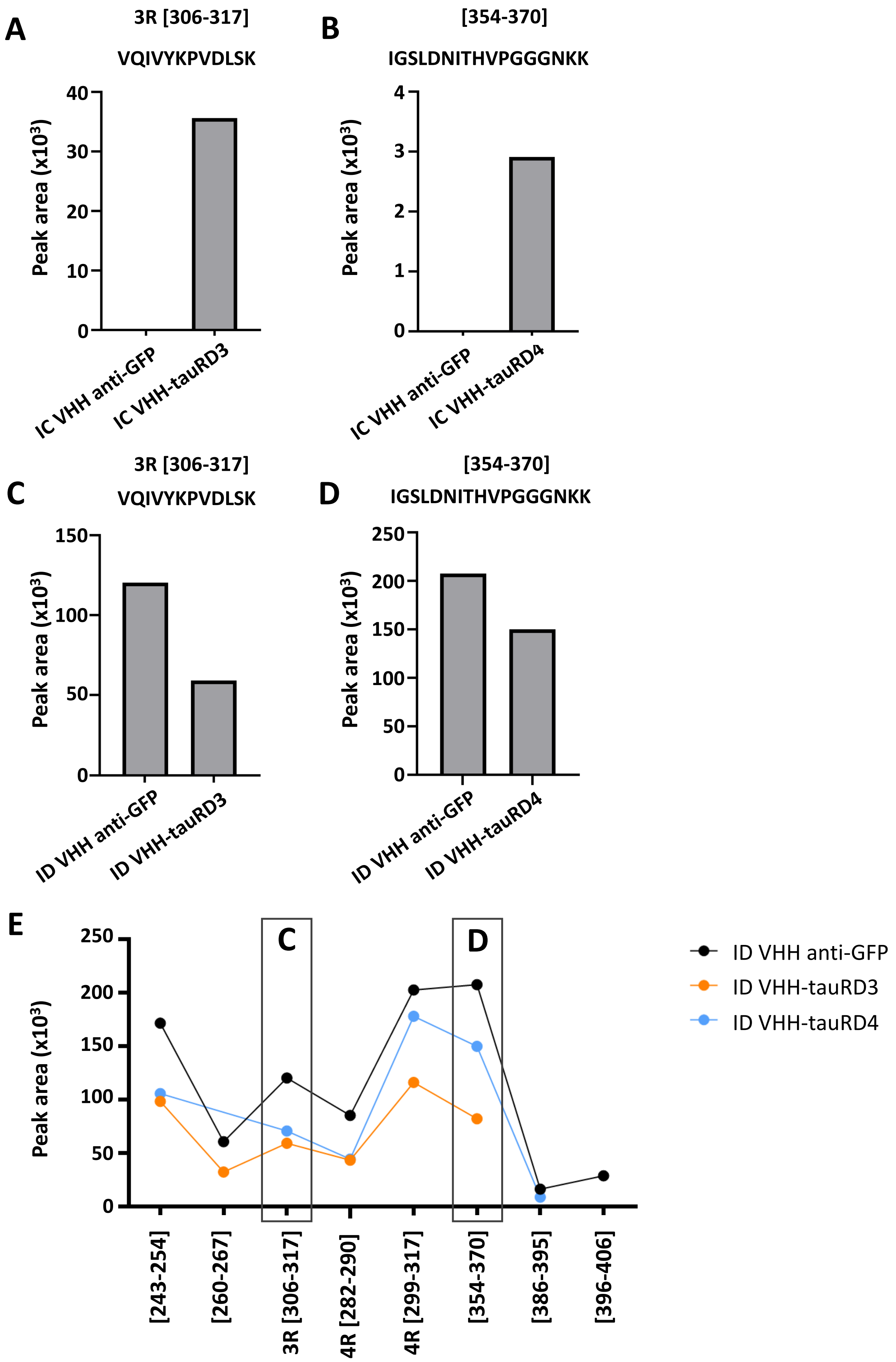

Supplementary figure 5: **Mass spectrometry validation of the immunocapture and immunodepletion protocol using VHH on BD-EVs.** The immunocapture of tau from $4x10^{10}$ BD-EVs from a pool of three AD patients was done, after which the IC and ID fractions were analyzed by mass spectrometry to assess the captured or removed tau tryptic peptides, respectively. **(A)** The tryptic peptide VQIVYKPVDLSK lies within the epitope of VHH-tauRD3. We observe an enrichment of this peptide compared to VHH anti-GFP. **(B)** The tryptic peptide IGSLDNITHVPGGGNKK lies within the epitope of VHH-tauRD4. We observe an enrichment of this peptide compared to VHH anti-GFP. **(C-D)** The ID fraction shows a reduction in tryptic peptide sequence VQIVYKPVDLSK using VHH-tauRD3 **(C)** and IGSLDNITHVPGGGNKK using VHH-tauRD4 **(D)** compared to VHH anti-GFP. **(E)** Line graph showing the different tryptic peptides detected in the ID fraction using VHH anti-GFP (black), VHH-tauRD3 (orange) and VHH-tauRD3 (blue). N=1.

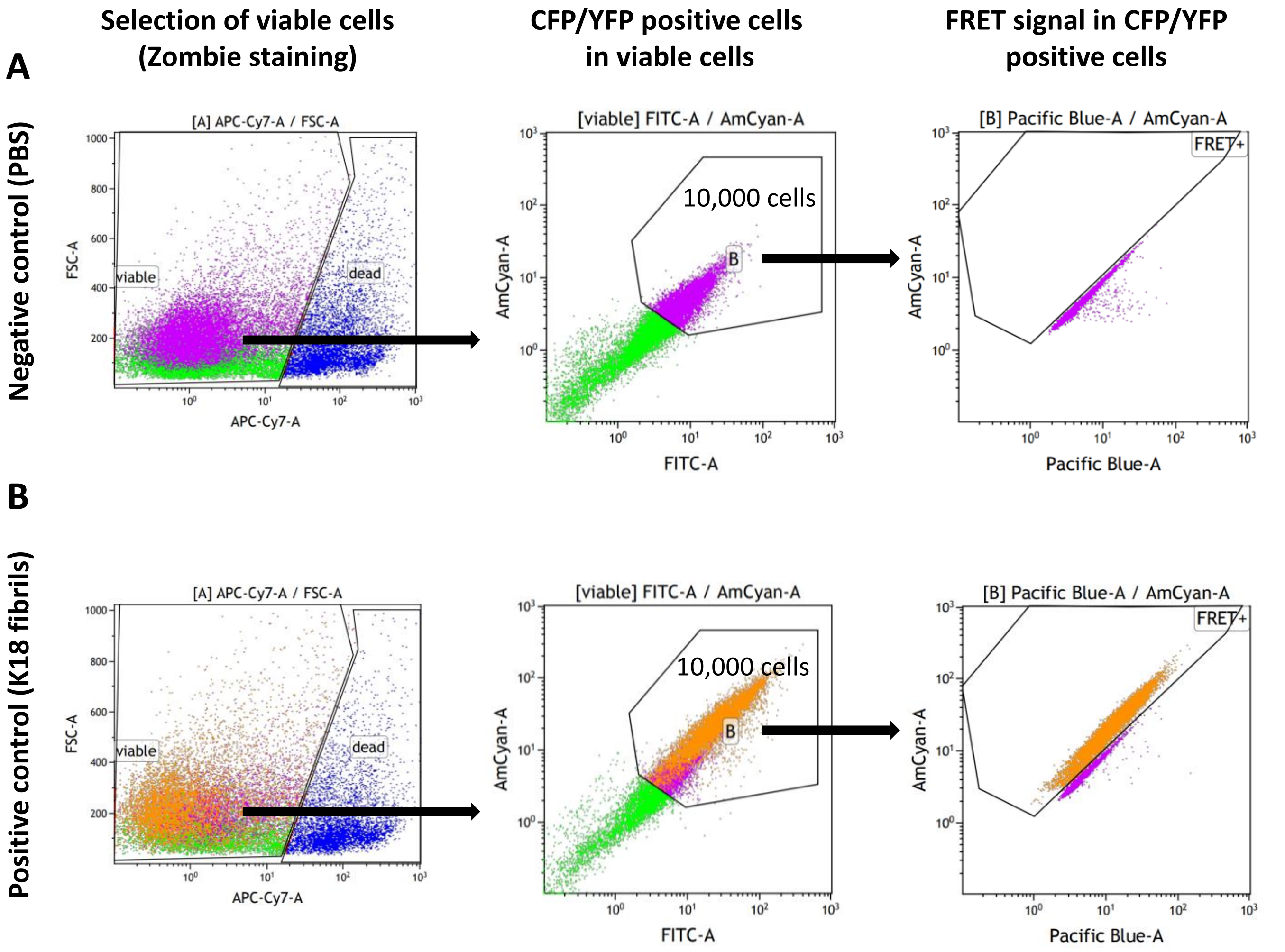

Supplementary Figure 6: **Gating strategy for the FRET–tau biosensor cell assay.** HEK FRET–tau biosensor cells were plated and lipofected 24 h later. Cells were collected 72 h post-lipofection and prepared for flow cytometry analysis by Zombie NIR staining followed by paraformaldehyde (PFA)-based fixation. This assay is used to quantify tau seeding activity. Gates were initially set using the flow cytometer software and further refined during post-analysis in Kaluza software, based on the negative control (PBS) included in each experiment. The first gating step excluded debris (not shown). Live cells were identified by the absence of Zombie NIR staining. This gating strategy is illustrated for PBS (negative control, **A left**) and sonicated K18 tau fibrils (2 µM; positive control, **B left**). Next, cells double-positive for tau-YFP and tau-CFP were selected (named gate B), as tau aggregation -and thus FRET signal generation- can only occur in this population. Acquisition was stopped after recording 10,000 YFP^+/CFP^+ events. Representative plots are shown for PBS (**A middle**) and K18 tau fibrils (**B middle**). Next, within the YFP^+/CFP^+ population, the percentage of FRET-positive (named gate $FRET^+$, shown in orange) cells was quantified. No $FRET^+$ cells were detected in PBS-treated samples after a post-analysis refinement of gates in Kaluza software to exclude false positive cells (**A right**), whereas a substantial $FRET^+$ population was observed following treatment with K18 tau fibrils (**B right**), reflecting tau aggregation.

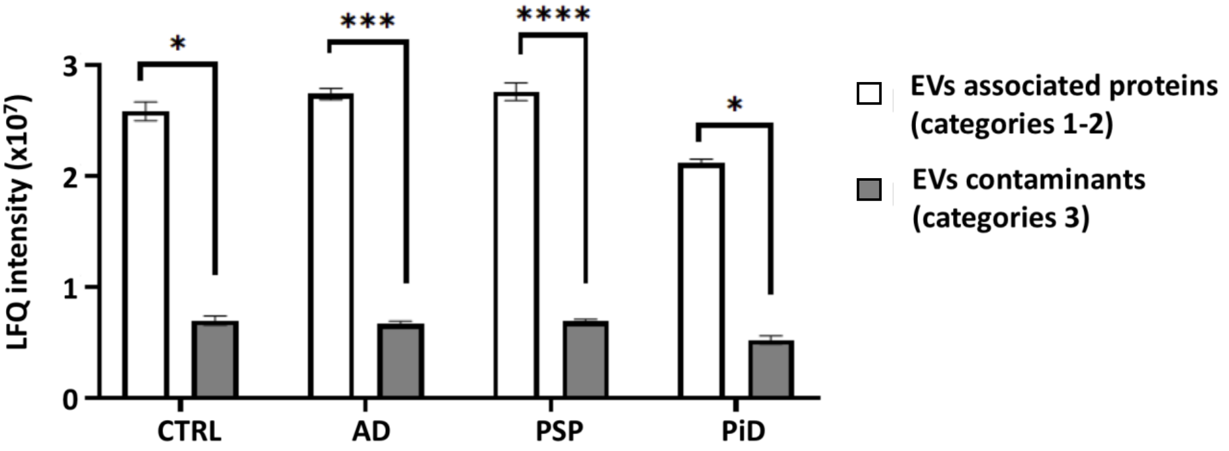

Supplementary figure 7: **Validation of BD-EVs purity based on proteomic data.** $4x10^9$ BD-EVs from the human cohort of CTRL (n=4), AD patients (n=8), PSP patients (n=9) and PiD patients (n=4) were prepared using the SP3 automated protocol on the LT Bravo Liquid handler (Agilent technologies) as described in Müller et al., 2020 (PMID: 32129943). Mass spectrometry data was generated by LC-MS/MS analysis with the EvoSep One liquid chromatography system coupled to the Q-TOF system (timsTOF HT, Bruker). The proteomic data reveals the BD-EVs content, which was crossed with the MISEV2023 guidelines to assess the purity of the samples. Hence, the vertical bar graph corresponds to the cumulative LFQ intensities of proteins associated with EVs or EVs contaminants. EVs associated proteins include categories; 1a: Multi-pass transmembrane proteins associated with plasma membrane and/or endosomes; 1b: Single-pass transmembrane proteins associated with plasma membrane and/or endosomes; 1c: GPI-or lipid-anchored proteins associated with plasma membrane and/or endosomes; 2a: Cytosolic proteins with lipid or membrane protein-binding ability; 2b: Cytosolic proteins with promiscuous incorporation into EVs. EVs contaminant proteins categories: 3a: Lipoproteins; 3b: Protein and protein/nucleic acid aggregates and 3c: Exomere or supermere-enriched components. Other categories were not included, as their association with BD-EVs remains putative. Multiple Mann-Whitney test, non-parametric comparison of EVs associated proteins and EVs contaminants for each tauopathy individually, *$p < 0.05$, ***$p < 0.001$, ****$p < 0.0001$.

**Total tau sequence recovery**	
2N4R tau amino acids range	[1-411]
Total number of 2N4R tau amino acids	441
Sum of non-detected amino acids	187
Percentage of non-detected amino acids	42.4%
Percentage of detected amino acids (sequence recovery)	57.6%

**N-domain sequence recovery**	
N-domain amino acids range	[1-102]
Total number of N-domain amino acids	102
Sum of non-detected amino acids of N-domain	6
Percentage of non-detected amino acids N-domain	10.2%
Percentage of detected amino acids (sequence recovery) N-domain	89.8%

**MTBR sequence recovery**	
MTBR amino acids range	[244-368]
Total number of MTBR amino acids	125
Sum of non-detected amino acids of MTBR	63
Percentage of non-detected amino acids MTBR	50.4%
Percentage of detected amino acids (sequence recovery) MTBR	49.6%

**C-terminal domain sequence recovery**	
C-terminal domain amino acids range	[369-441]
Total number of C-terminal domain amino acids	73
Sum of non-detected amino acids of C-terminal domain	42
Percentage of non-detected amino acids C-terminal domain	57.5%
Percentage of detected amino acids (sequence recovery) C-terminal domain	42.5%

Supplementary figure 8: **Tau sequence recovery after tau-enriched mass spectrometry analysis.** Table indicating the percentage of tau sequence recovery for: full-length 2N4R (57.6%), N-terminal domain (89.8%), MTBR (49.6%) and C-terminal domain (42.5%) after the immunocapture of tau from EVs using anti-TauE1C1, anti-TauP1 and anti-Tau7F5, followed by mass spectrometry analysis.

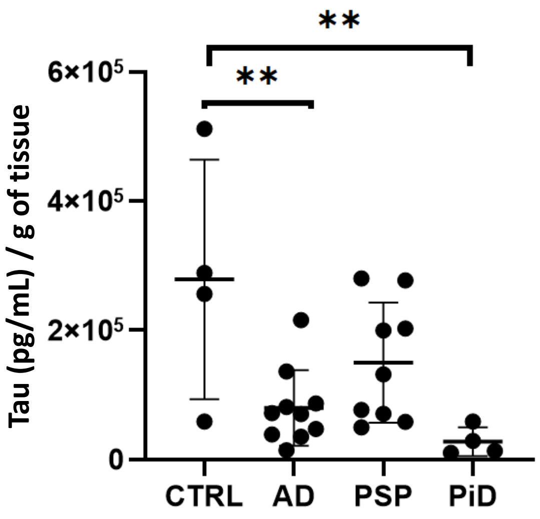

Supplementary figure 9: **Simoa quantification of total tau in BD-EVs.** Dotplot indicating the tau levels expressed in pg/mL normalized to the quantity of brain tissue used to prepare the prefrontal BD-EVs of CTRL, AD, PSP and PiD. One-way ANOVA, parametric multiple comparison between all groups, **$p < 0.01$.

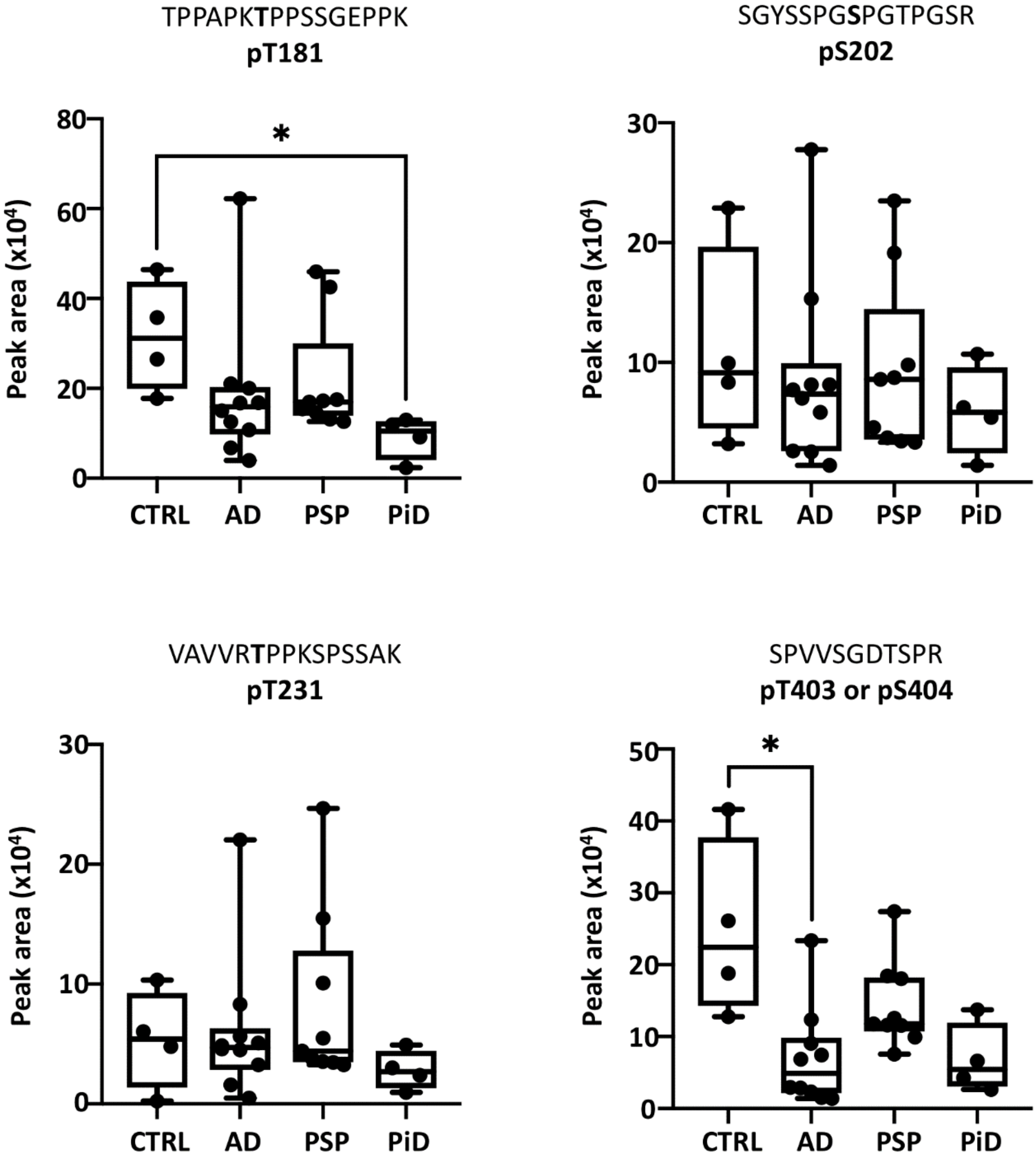

Supplementary figure 10: **Comparison of tau tryptic peptides with a phosphorylation.** BD-EVs from the human cohort of CTRL (n=4), AD patients (n=8), PSP patients (n=9) and PiD patients (n=4) were isolated and lysed. After which a tau enrichment IP-MS was done. Four tau phospho-peptides with one or two possible phosphorylation sites were detected in BD-EVs. Boxplots expressed as peak area of the light tau peptides. Kruskal-Wallis, non-parametric multiple comparison between CTRL, AD, PSP and PiD for each tryptic peptides, *$p < 0.05$.

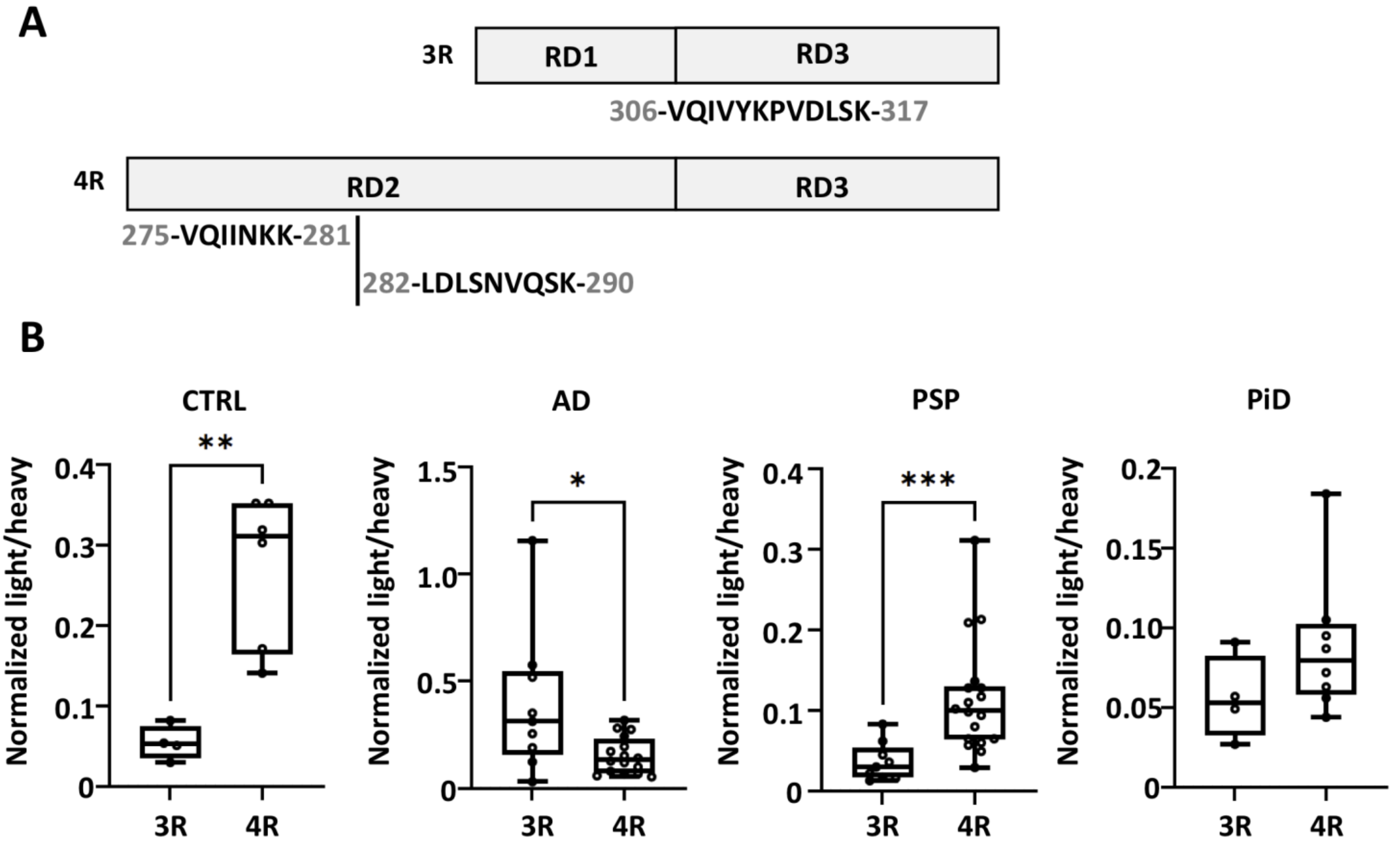

Supplementary figure 11: **3R- and 4R- isoform specific peptides in BD-EVs. (A)** Schematic overview of 3R-specific VQIVYKPVDLSK[306-317] tryptic peptide and the 4R specific tryptic tau peptides VQIINKK[275-281] and LDLSNVQSK[282-290]. **(B)** Boxplots of the normalized peak area of the light-over-heavy isotope for controls, AD, PSP and PiD. 4R-specific tryptic peptides were pooled for comparison with 3R specific tau. Unpaired t-test, parametric comparison between 3R and 4R specific peptides of controls, AD, and PiD, *p < 0.05, **p < 0.01. Mann-Whitney test, non-parametric comparison between 3R and 4R specific peptides of PSP, ***p < 0.001.

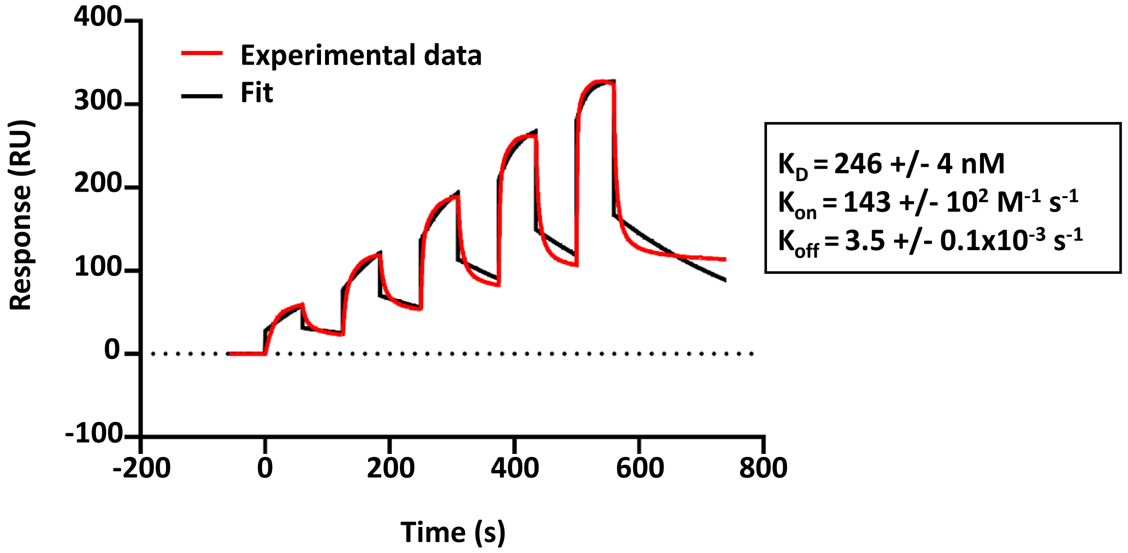

Supplementary figure 12: **Affinity determination of VHH-tauRD3 binding to tau2N4R by SPR**. Affinity measurements were performed at 25°C on a BIAcore T200 optical biosensor instrument (Cytiva). Capture of biotinylated tau2N4R in HBS EP+ buffer (Cytiva) was performed on a SA sensor chip (Cytiva), until the total amount of captured proteins reached 300 resonance units. A flow cell was used as a reference to assess non-specific binding and to allow for background correction. VHH Z70 (VHH-tauRD3) was injected sequentially at increasing concentrations ranging between 0.25 and 4 µM in a single-cycle at a flow rate of 30 µL/min. Sensorgrams (reference subtracted data) of single cycle kinetics analysis performed on immobilized biotinylated tau2N4R, with five injections of increasing concentrations of VHH-tauRD3. k_{on} and k_{off} and K_D values are included in the figure. Black lines correspond to the fitted curves, red lines correspond to the measurements.
